# Regulatory modules mediating the complex neural expression patterns of the *homeobrain* gene during *Drosophila* brain development

**DOI:** 10.1186/s41065-021-00218-5

**Published:** 2022-01-05

**Authors:** Kirsten Hildebrandt, Dieter Kolb, Christine Klöppel, Petra Kaspar, Fabienne Wittling, Olga Hartwig, Jannic Federspiel, India Findji, Uwe Walldorf

**Affiliations:** 1 Developmental Biology, Saarland University, Building 61, 66421 Homburg/Saar, Germany; 2 Present address: COS Heidelberg, University of Heidelberg, Im Neuenheimer Feld 230, 69120 Heidelberg, Germany; 3 Present address: Hemholtz Institute for Pharmaceutical Research Saarland (HIPS), Saarland University, Building E8.1, 66123 Saarbrücken, Germany

**Keywords:** Homeobrain (Hbn), Transcription factor, Enhancer, Gene targeting

## Abstract

**Background:**

The homeobox gene *homeobrain* (*hbn*) is located in the 57B region together with two other homeobox genes, *Drosophila Retinal homeobox* (*DRx*) and *orthopedia* (*otp*). All three genes encode transcription factors with important functions in brain development. *Hbn* mutants are embryonic lethal and characterized by a reduction in the anterior protocerebrum, including the mushroom bodies, and a loss of the supraoesophageal brain commissure.

**Results:**

In this study we conducted a detailed expression analysis of Hbn in later developmental stages. In the larval brain, Hbn is expressed in all type II lineages and the optic lobes, including the medulla and lobula plug. The gene is expressed in the cortex of the medulla and the lobula rim in the adult brain. We generated a new hbn^KOGal4^ enhancer trap strain by reintegrating Gal4 in the *hbn* locus through gene targeting, which reflects the complete *hbn* expression during development. Eight different enhancer-Gal4 strains covering 12 kb upstream of *hbn*, the two large introns and 5 kb downstream of the gene, were established and *hbn* expression was investigated. We characterized several enhancers that drive expression in specific areas of the brain throughout development, from embryo to the adulthood. Finally, we generated deletions of four of these enhancer regions through gene targeting and analysed their effects on the expression and function of *hbn*.

**Conclusion:**

The complex expression of Hbn in the developing brain is regulated by several specific enhancers within the *hbn* locus. Each enhancer fragment drives *hbn* expression in several specific cell lineages, and with largely overlapping patterns, suggesting the presence of shadow enhancers and enhancer redundancy. Specific enhancer deletion strains generated by gene targeting display developmental defects in the brain. This analysis opens an avenue for a deeper analysis of *hbn* regulatory elements in the future.

## Background

The *Drosophila* nervous system arises from a relatively small number of neural lineages compared with higher organisms. In the embryo, approximately 80 bilateral symmetric lineage pairs eventually form the ventral nerve cord and the suboesophageal ganglion, and the central brain is formed by 108 bilaterally arranged lineages [1–4]. Each lineage derives from a stem cell called a neuroblast that divides asymmetrically and thereby generates a further neuroblast and a neuronal precursor cell, the ganglion mother cell (GMC) through self-renewal. The GMC subsequently divides symmetrically and produces two neurons. Through this mode of division, the neuroblast produces embryonic lineages of primary neurons [5]. This type of division is typical for type I neuroblasts that comprise most of the cell lineages in the embryonic brain. In contrast to type I neuroblasts, type II neuroblasts generate intermediate neural progenitor cells (INPs) that divide several times to generate GMCs, which in turn divide into two neurons [6–8], thereby generating larger lineages. Eight of these type II neuroblasts and the corresponding lineages are also observed in later stages of embryonic brain development [9, 10]. At the end of embryogenesis, most neuroblasts undergo a period of quiescence and resume their division during the larval stage [11]. Embryonic neuroblasts account for only 10% of the adult neurons, whereas divisions after the quiescent period generate the remaining 90% of the adult neurons [12]. In the larval brain, all neuroblasts generate larger lineages compared to the embryonic brain, type I lineages produce a progeny of 100 neurons, and type II lineages produce up to 400 neurons [12]. The type I and type II lineages build the central brain region of the larval brain hemispheres and are flanked by the optic lobes, whose cells are derived from the optic placodes already present in the embryo and are closely associated with the brain. These cells grow during the larval stages [13] and through complex morphogenetic movements, they build the larval optic lobes with four neuropil structures: the medulla, the lamina and the lobula complex, consisting of the lobula and lobula plug ([14] for review). These larval structures produce the adult structures medulla, lamina lobula and lobula plate respectively, through changes in position and orientation, which are already well described [15]. Lineages of the larval central brain generate well-known adult brain structures such as the antennal lobes, mushroom bodies and the large central complex. In particular, the central complex is of great interest as an integration centre for motor, sensory, learning and memory activities ([16] for review).

Several transcription factors were identified that are important for the proliferation of type I and type II neuroblasts in the brain, leading to an expansion of the brain region compared with the ventral nerve cord [17]. They include Earmuff (Erm), belonging to the FEZ family of C2H2 Zinc finger transcription factors [18], Tailless (Tll), a nuclear receptor transcription factor [19], the T-box transcription factors Doc1/2/3 [20] as well as the three homeodomain transcription factors Orthopedia (Otp) [21, 22], Drosophila Retinal homeobox (DRx) [23, 24] and Homeobrain (Hbn) [25, 26], clustered in the 57B region on the second chromosome. Mutants of all these factors alone or in combination show a reduction of neuroblasts and the proliferation of their daughter cells in the embryonic brain. Upon misexpression, all of these transcription factors drive proliferation in the ventral nerve cord and even reprogram wing disc cells into brain neural progenitors [17]. The homeodomain transcription factor Hbn, which is one of these brain factors, was shown to be expressed in the embryo in several neuroblasts, in GMCs and in neurons located mainly in the protocerebrum [26]. These neuroblasts include type I neuroblasts and some type II neuroblasts [17].

In *hbn* mutants, large parts of the protocerebrum are missing and the supraesophageal commissure connecting both brain hemispheres is absent [26]. In addition, Hbn is expressed in the midgut of late embryos [25], with an apparent endocrine function. Single-cell RNA-sequencing recently revealed that a transcription factor code for enteroendocrine cells exists in the adult *Drosophila* midgut [27]. Here, 10 different enteroendocrine subtypes produce 14 different classes of hormone peptides. Hbn is part of this transcription factor code and functions as a transcriptional repressor of the hormones Allatostatin A and Diuretic hormone 31 [27].

The complex expression of Hbn in specific lineages during embryonic brain development also suggests later expression and a distinct function in postembryonic stages. Accordingly, RNA-seq data indicate the expression of Hbn throughout all larval and pupal stages up to the adult stage [28]. In the larval brain, a series of transcription factors is expressed over time in the dorsomedial (DM) lineages and in the medulla, endowing the respective cells with specific temporal identities [29–31]. These time series factors include Dichaete (D), Grainyhead (Grh) and Eyeless (Ey) expressed in the DM lineages [29] and Homothorax (Hth), Klumpfuss (Klu), Eyeless (Ey), Sloppy paired (Slp), Dichaete (D) and Tailless (Tll) expressed in the optic lobe [30, 31]. Hbn was recently identified as another factor of this time series that is expressed in an overlapping pattern with Eyeless right before Sloppy paired [32] and interacts with both of these transcription factors to mediate activation and repression [33].

One major question is how the complex expression patterns of Hbn are generated. Researchers have accepted that the expression of genes in specific domains or tissues is regulated by sets of regulatory elements, among which enhancers act over large distances. Reporter gene assays are usually performed using lacZ or GFP as reporter genes to analyse such elements in *Drosophila* ([34] for review). In the course of the *Drosophila* genome project, more systematic efforts were attempted to identify gene enhancers. In one of these approaches, putative enhancer regions of genes with a known expression or function in the adult brain were analysed [35]. For this project, overlapping pieces of 3 kb upstream, downstream or in intronic regions of the respective 925 genes were cloned in front of a Gal4 gene, transgenic fly strains were established, and the expression pattern of putative enhancers was analysed in different developmental stages and tissues using reporter genes [36–38].

In addition to the identification of specific enhancers for a gene of interest, a functional analysis of enhancers was performed by downregulating of a gene with RNAi driven by a specific enhancer using the UAS/Gal4 system [39]. As mentioned above, one would expect a downregulation, but maybe not a complete loss of activity due to a temporal delay in the inactivation, which in most cases is not 100%. Using other techniques, such as ends-out gene targeting [40–42], or using the CRISPR/Cas9 system [43, 44] which also functions in *Drosophila* [45–47], researchers can delete enhancers that would produce stronger phenotypes and much more reliable results.

In this paper we focus on the expression of Hbn in postembryonic stages and analyse the regulation of Hbn expression during brain development. Our analysis shows that Hbn is expressed in all type II lineages, some type I lineages, the mushroom bodies, the medulla, and the lobula plug in the larval brain, and in the medulla and lobula rim of the adult brain. In the larval type II lineages, Hbn is expressed in INPs and in GMCs and neurons, but not in glial cells. Through gene targeting, we generated a new *hbn* allele with a partial deletion of the first exon including the ATG start codon. Gal4 was reintegrated at that position to generate a *hbn* enhancer trap strain that was subsequently analysed. Moreover, the entire upstream and downstream region of the *hbn* gene, as well as the two largest introns were scanned for potential regulatory elements. To this end, eight Gal4 strains harbouring the various enhancer fragments were generated and tested for expression. We identified several regulatory modules responsible for the complex expression of Hbn in the embryonic brain, in type II lineages, mushroom bodies, the medulla and lobula plate in the larval brain and in the medulla and lobula rim of the adult brain. In our final analysis, four enhancer regions driving prominent expression in the type II lineages and the optic lobe were individually deleted by gene targeting to analyse the effects on Hbn expression. Our findings reveal important functions of Hbn in various processes of *Drosophila* brain development.

## Results

### Hbn expression during *Drosophila* development

**Fig. 1:**
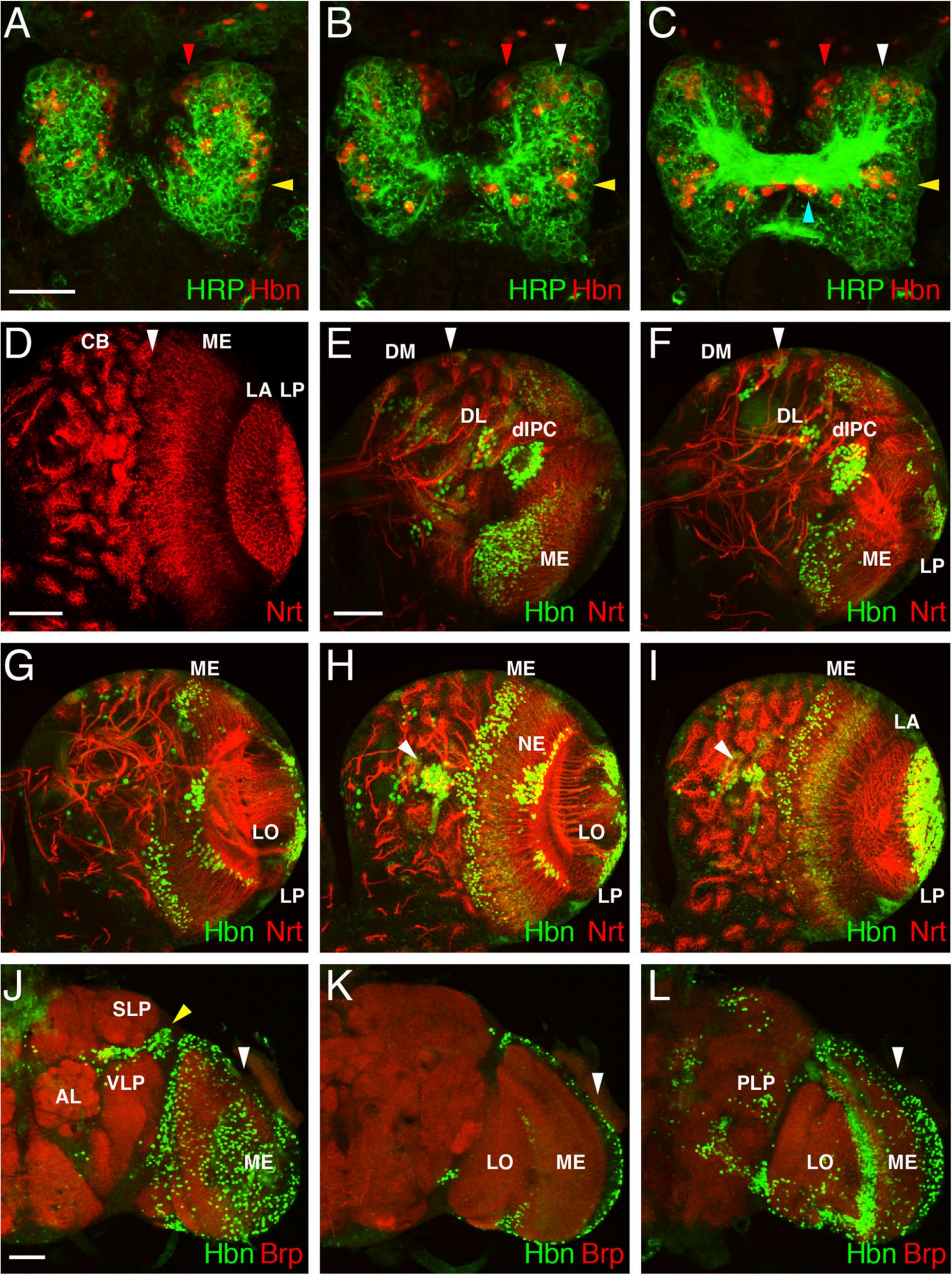
Hbn expression during *Drosophila* development. Laser confocal images of *Drosophila* embryonic, larval and adult brains. **A-C** Images of HRP (green) and Hbn (red) staining in three sections of an embryonic brain at stage 15 from dorsal to the ventral (brain commissure level). Major expression domains are indicated by arrowheads, including the medial region (red arrowheads), central region (white arrowheads), lateral region (yellow arrowheads), and brain commissure (blue arrowhead). **D** Ventral view showing Nrt staining in a right larval brain hemisphere (L3) to highlight the main structures. CB, central brain with type I cell lineages; ME, medulla; LA, lamina; LP, lobula plate. The border between the central brain region and the optic lobe region is indicated by a white arrowhead. **E-I** Dorsal to ventral sections of a right larval brain hemisphere stained with anti-Hbn (green) and anti-Nrt (red) antibodies. The largest expression domains are indicated: the dorsomedial domain (DM), dorsolateral domain (DL), the dorsal inner proliferation centre (dIPC), the medulla (ME), the neuroepithelium (NE), the lobula (LO), the lobula plate (LP) and the ventral central brain domain (white arrowhead). No expression is observed in the lamina (LA). Additional smaller domains are also indicated by white arrowheads. **J-L** Images of three different focal planes of the right part of an adult brain showing the expression of Hbn in green and Brp in red. **J** In the more anterior focal plane, Hbn expression is observed in the medulla (ME) and a discrete domain between VLP and SLP (yellow arrowhead). **K** In a more medial focal plane Hbn expression is also observed in the medulla (ME) (white arrowhead). **L** In the posterior focal plane, Hbn is expressed in the medulla (ME) (white arrowhead) and some cells in the central brain. Abbreviations: AL, antennal lobe; LO, lobula; PLP, posterior-lateral protocerebrum; SLP, superior-lateral protocerebrum; VLP, ventro-lateral protocerebrum. (Scale bars: **A-C**, 25 μm; **D**, 50 μm; **E-I**, 50 μm as in **E**; **K-M**, 50 μm as in **K**)

During embryonic brain development, Hbn is expressed mainly in protocerebral lineages of medial, central and lateral subareas dorsally and ventrally, including neuroblasts, GMCs and neurons. *Hbn* mutants are embryonic lethal and are characterized by severe defects in the protocerebrum, where the supraesophageal commissure and parts of the anterior protocerebrum, including the mushroom bodies, are lost through apoptosis [26]. These findings revealed an important function of Hbn in embryonic brain development. For an overview and comparison, three sections of an embryonic brain from a stage 15 embryo stained for Hbn in combination with HRP as a general neuronal marker for sensory neurons, peripheral nerves and all fibre tracts [48] are shown here (Fig. 1A-C). The different expression domains of Hbn in the medial, central, and lateral cells and those associated with the commissure are marked (Fig. 1A-C, red, white, yellow and blue arrowheads, respectively).

Next, we determined Hbn expression during larval development and in adults, stages that were not yet analysed. The *Drosophila* larval brain consists of a ventral nerve cord and two brain lobes. The major areas of a brain lobe are shown in Fig. 1D using Neurotactin (Nrt) [49], a marker that is expressed in many postembryonic secondary neurons and their axons. Each brain lobe has a central brain (CB) region with the mushroom bodies and various cell lineages, here type I lineages, and a more lateral region, the optic lobe, subdivided in the medulla (ME), lamina (LA) and lobula plate (LP) from medial to lateral [50] (Fig. 1D). Cells of the optic lobe are derived from two proliferation centres: an outer proliferation centre (OPC) generating the medulla and lamina and an inner proliferation centre (IPC) generating mainly cells of the lobula complex [51]. During larval development, Hbn is expressed in discrete areas of the larval brain. In dorsal views expression was detected in the six type II dorsomedial (DM) lineages and the two type II dorsolateral (DL) lineages (Fig. 1E, F) and an additional smaller domain (white arrowheads). In the optic lobe region, expression was observed in the dorsal inner proliferation centre (dIPC) and in the medulla (ME) (Fig. 1E, F). In more medial and ventral sections, Hbn was expressed in the medulla (ME) in the neuroepithelium (NE) and the neuropil (Fig. 1G-I), as well as in the lobula (LO) (Fig. 1G, H) and lobula plate (LP) (Fig. 1F-I), whereas no Hbn expression was detected in the lamina (LA) (Fig. 1I). In ventral sections of the central brain, expression was observed in a ventral brain lineage (Fig. 1H, I, white arrowheads).

In the adult brain, Hbn expression was analysed in combination with an antibody against Bruchpilot (Brp) which labels synapses and is used to mark the neuropil [52]. In a more anteriorly located focal plane, the antennal lobe (AL) was clearly visible (Fig. 1J). In the nearby regions, Hbn was expressed between the ventro-lateral protocerebrum (VLP) and the superior-lateral protocerebrum (SLP) (Fig. 1J, yellow arrowhead). Most expression was detected in the medulla (ME) (Fig. 1J, white arrowhead). In more posterior focal planes, prominent expression was again observed in the medulla (ME) (Fig. 1K, L, white arrowheads), but not in the lobula (LO). Some scattered expression was detected in the region of the posterior-lateral protocerebrum (PLP) and a few other regions in the central brain.

**Fig. 2:**
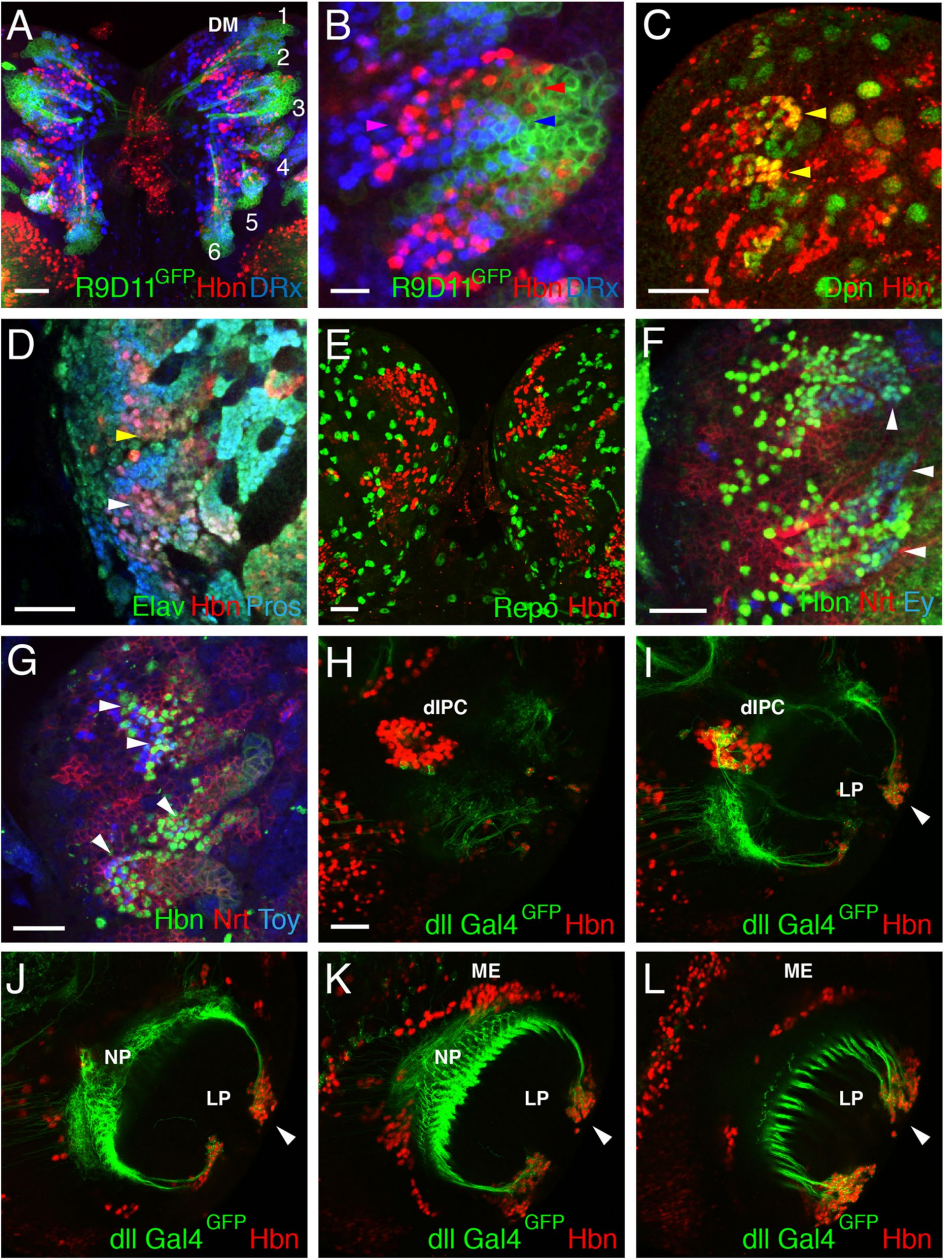
Cell type identification of Hbn expressing cells in the larval brain. Images of larval brains showing the central brain regions (**A**, **E**) or brain hemispheres (**B-D**, **F-L**). **A** The earmuff R9D11 reporter (R9D11-mCD8-GFP, green) highlights the proximal parts of the six DM lineages (1–6) starting from the intermediate neural progenitor cells (INPs). DRx expression (blue) is observed in the more distal regions of each lineage compared with Hbn expression (red) in the more medial and proximal parts of these lineages. **B** A higher magnification image of some DM lineages shows that Hbn expression (red) in the proximal and medial regions is located more anteriorly (red arrowhead), and DRx expression (blue) in the medial and distal parts is located more posteriorly within the lineage (blue arrowhead). A region of coexpression of both proteins also exists (magenta arrowhead). **C** Dpn expression (green) marks INPs of type II lineages. Coexpression with Hbn (red) is observed in some cells of each lineage (yellow arrowheads). **D** The expression of Hbn (red) in combination with Pros (blue) and Elav (green) indicates Hbn expression in GMCs (Pros^+^, Elav^−^, white arrowhead) and neurons (Pros^+^, Elav^+^, yellow arrowhead). **E** Hbn (red) is not coexpressed with the glial cell marker Repo (green). **F** Hbn expression (green) overlaps with the DM time series marker Ey (blue) in some INPs (white arrowheads). **G** Toy (blue) is expressed more distally in neurons of the DM lineages and overlaps with Hbn (green) in a few cells (white arrowheads). (**H-L**) Sections of a larval brain hemisphere focusing on the lobula complex. Hbn expression (red) is shown in comparisons with dll-Gal4-mCD8::GFP (green). Abbreviations: DM, dorsomedial lineages; dIPC, dorsal inner proliferation centre; LP, lobula plate; ME, medulla; NP, neuropil. (Scale bars: 25 μm, 2B 10 μm)

We used the Gal4 line Erm-Gal4-R9D11 [35] to analyse the expression of Hbn in the type II lineages in more detail. This line specifically drives Gal4 expression in the proximal parts of the DM lineages in intermediate neural progenitor cells (INPs) and ganglion mother cells (GMCs) [18, 53, 54]. Erm-Gal4 expression was visualized using a UAS-mCD8::GFP reporter (membrane staining); it started in neuroblasts but was also present in INPs and GMCs (Fig. 2A). As additional marker expressed in the DM lineages, we used DRx [55], which is expressed in many cells from each lineage ranging from the medial part of the lineage to the most distal part. Compared to DRx, Hbn was expressed in fewer cells in each lineage ranging from the medial part to a more proximal region (Fig. 2A), which was more visible in a higher magnification image (Fig. 2B). Whereas Hbn was in generally expressed in the anterior part within each lineage (Fig. 2B, red arrowhead), DRx was expressed more posteriorly and distal (Fig. 2B, blue arrowhead). Coexpression in the central region of the lineage was also visible (Fig. 2B, magenta arrowhead). The Hbn-expressing cells in the proximal part of each lineage were most likely INPs. We performed stainings to detect Hbn and Deadpan (Dpn), a marker for neuroblasts and INPs [56], to determine whether our hypothesis was valid and to identify the other cells in which Hbn was also expressed. In some cells, both proteins were coexpressed, indicating that Hbn is at least expressed in mature INPs (Fig. 2C, yellow arrowheads). The expression of Hbn in GMCs and neurons was analysed using the markers Prospero and Elav. Prospero is expressed in the cytoplasm of neuroblasts, in the nucleus of GMCs after asymmetric division, and later also in neurons [57, 58], whereas Elav is only expressed in postmitotic neurons [59, 60]. Therefore, an Elav-negative cell with Prospero nuclear staining indicates a GMC, and an Elav-positive cell indicates a neuron. Hbn expression was clearly detectable in some GMCs (Fig. 2D, white arrowhead) and many neurons (Fig. 2D, yellow arrowhead). To assay Hbn expression in glial cells, Reversed polarity (Repo) was used as a general glial cell marker [61, 62]. However, no colocalization of Repo with Hbn was detected in the dorsomedial lineages (Fig. 2E). In summary, in the dorsomedial lineages, Hbn is expressed in the more proximal and anterior regions in INPs, GMCs and neurons, but not in glial cells.

We performed staining of Hbn together with Eyeless (Ey) and Twin-of-eyeless (Toy) to compare the expression of Hbn with some of the known timing factors of the DM lineages [29]. Eyeless was expressed proximal in the DM lineages in INPs and showed overlap with some of the Hbn expressing cells (Fig. 2F, white arrowheads). In contrast, Toy was expressed more distally in the lineages in neurons, and overlapping with Hbn in just a few cells (Fig. 2G, white arrowheads). The expression of Toy extended more distally than Hbn expression.

The expression of Hbn in the medulla was not analysed further, since a paper submitted recently [33] explores this topic. However, in addition to its expression in the medulla, Hbn is also expressed in the lobula complex. We analysed lobula plate expression more specifically using the marker dll-Gal4 to visualize the neurons of the lobula plate and their axonal projections [63, 64]. Optical sections of a brain hemisphere showed Hbn expression in the dorsal inner proliferation centre (dIPC) (Fig. 2H, I), the adjacent medulla (ME) (Fig. 2K, L) and the distal region of the lobula plate (LP) (Fig. 2I-L, white arrowheads).

### Generation of an *hbn* mutant strain with a reintegration of Gal4 in the *hbn* locus

**Fig. 3:**
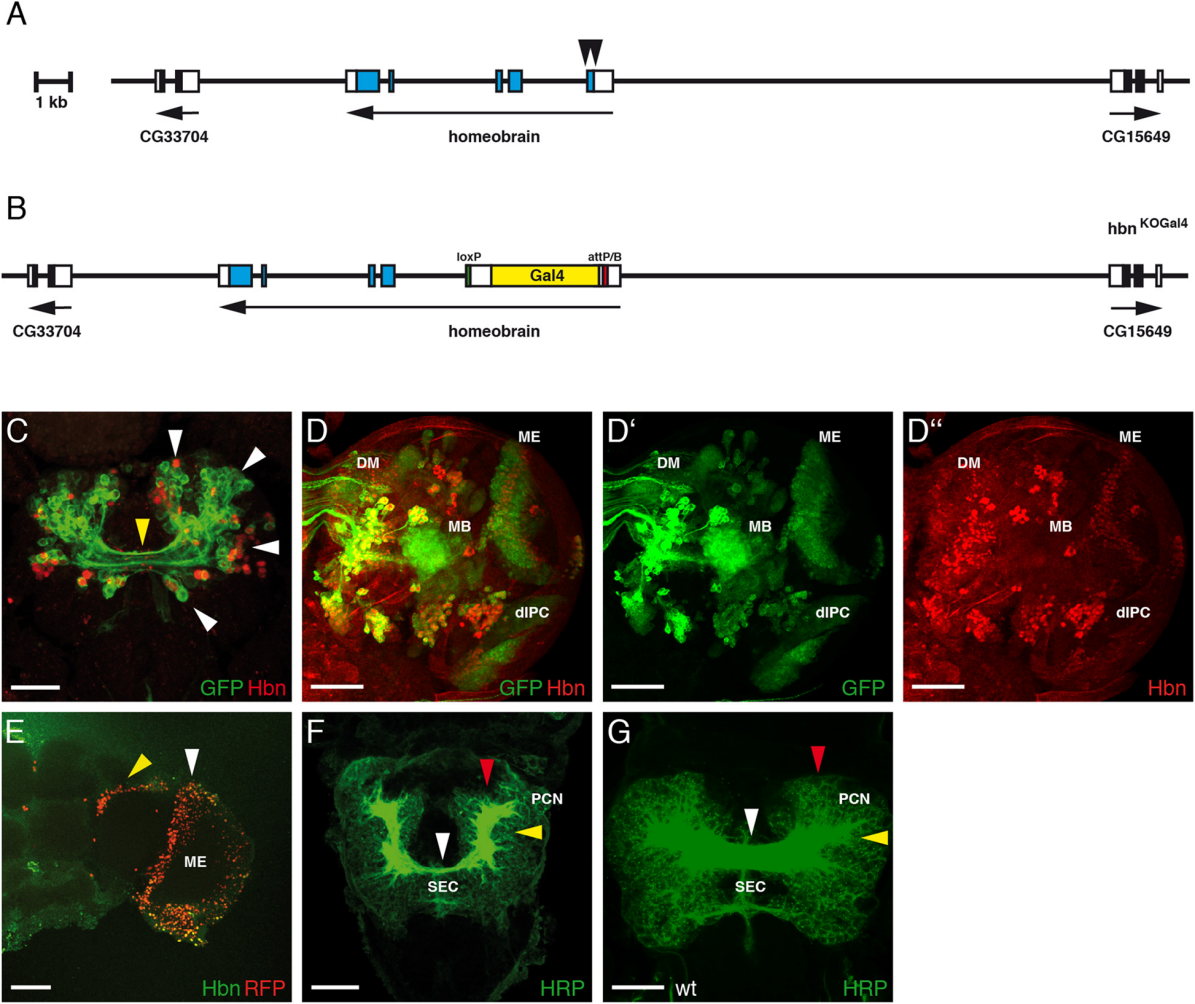
Generation and expression of the hbn^KOGal4^ strain. **A** The genomic organization of the *hbn* locus is shown with the positions of the five exons [25] relative to the next upstream and downstream genes (CG15649 and CG33704). Noncoding regions are indicated by white boxes, and coding regions are indicated by blue boxes. The region deleted in the gene targeting construct is indicated by black arrowheads. **B** The genomic organization of the hbn^KOGal4^ strain is indicated. Here, the region upstream of the ATG in exon 1 up to sequences immediately downstream of the exon 1 donor splice site was deleted and replaced by Gal4 (yellow) flanked by an attP/B site (red) and a loxP site (green). **C-E** Laser confocal images show the expression in hbn^KOGal4^ heterozygous animals at different developmental stages visualized using a UAS-mCD8::GFP strain and a UAS-H2B-mRFP1 strain. **C** In a stage 16 embryonic brain, Hbn expression is shown in red, and hbn^KOGal4^ dependent marker GFP expression is shown in the membrane in green. Coexpression of GFP and Hbn is observed in several regions of the embryonic brain (white arrowheads), and axons crossing from one brain hemisphere to the other are also labelled (yellow arrowhead). **D** In the right hemisphere of a third instar larval brain, Hbn and the hbn^KOGal4^ marker coexpression is observed in the DM lineages, mushroom bodies (MB) and dorsal inner proliferation centre (dIPC). In the medulla (ME), the GFP marker is expressed in some regions alone (**D‘**) compared to Hbn expression (**D‘‘**). **E** In the right part of an adult brain coexpression of Hbn and RFP is detectable in the medulla (ME) and an additional domain between VLP and SLP (yellow arrowhead) (see Fig. 1J for comparison). **F** In a stage 16 homozygous hbn^KOGal4^ embryo stained with an anti-HRP antibody, the typical *hbn* mutant brain phenotype is visible. The size of the supraesophageal brain commissure (SEC) is substantially reduced (white arrowhead), the protocerebral connectives (PCN) are disorganized (yellow arrowhead) and the size of the anterior protocerebrum is reduced (red arrowhead) (**G**) A stage 16 wild-type embryo is shown for comparison. Abbreviations: DM, dorsomedial lineage; dIPC, dorsal inner proliferation centre; MB, mushroom bodies; ME, medulla. (Scale bars: **C**, **F**, **G**, 25 μm; **D-D‘‘**, **E**, 50 μm)

Based on the complex expression pattern of *hbn* during all stages of development, we postulated that an *hbn* enhancer trap strain with the possibility to misexpress or downregulate other genes in an *hbn*-dependent pattern might be a good tool for future experiments. We wanted to delete a part of the first exon, including the ATG start codon, through gene targeting to generate an *hbn* mutant allele followed by the integration of Gal4 at that position as a follow up on this idea. This strain could be used as an enhancer trap strain for *hbn* with the possibility of analysing *hbn* expression on the wild-type at all stages and in an *hbn* mutant background, at least in the embryo, since already known *hbn* alleles are embryonic lethal [26]. For the construction of this gene targeting construct, we used the vector pTV^cherry^ [65], which is suitable for this experimental design. We decided to delete a region of 171 bp starting 13 bp upstream of the ATG up to the first intron including the donor splice site (Fig. 3A, black arrowheads). We amplified and cloned two 4 kb homologous regions flanking the regions to be deleted in the pTV^cherry^ vector, generated transgenic fly lines and mapped their chromosomal positions. For the targeting event mediated by homologous recombination, we used a strain with an integration of the construct into the third chromosome that was not lethal to avoid negative effects if some P-element sequences remained at that position after recombination of the vector cassette in the *hbn* locus. Among the 18,000 flies produced as offspring of our gene targeting crosses, we identified 30 red-eyed flies, resulting in a targeting frequency of 1/600. Some of these flies were balanced and analysed using PCR to verify correct homologous recombination. In one of the final *hbn*-targeting strains, which we called hbn^KO^, sequences encoding ATG and the first 51 amino acids are replaced by a cassette including the *white* marker, loxP sites and an attP sequence [65]. This strain was embryonic lethal. Using the loxP sites, we removed the *white* gene and integrated Gal4 at the attP position with the help of the reintegration vector RIV^Gal4^ [65]. The *white* marker was removed using the flanking loxP sites so that in the final fly strain, Gal4 and some adjacent sequences replaced the deleted *hbn* exon sequences (Fig. 3B). We analysed this strain, which we called hbn^KOGal4^, by visualizing Gal4 expression with the help of the mCD8::GFP marker in the embryonic and larval brain (Fig. 3C, D) and the H2B-mRFP1 marker in the adult brain (Fig. 3E). In a stage 16 embryonic brain, the GFP marker was coexpressed in the cell membranes with Hbn in most regions where Hbn was expressed and several primary axon tracts (Fig. 3C, white arrowheads). GFP expression was also visible in axons composing the supraesophageal commissure, a structure that depends on Hbn expression (Fig. 3C, yellow arrowhead). In the larval brain, GFP marker expression was observed in the DM lineages, mushroom bodies (MB), dorsal inner proliferation centre (dIPC) and medulla (ME) (Fig. 3D). Most of these expression domains showed coexpression with Hbn, except for the medulla; here, the GFP marker was expressed at higher levels than Hbn due a longer perdurance of GFP compared to Hbn. This is better visible when GFP and Hbn expression are shown individually (Fig. 3D‘, D‘‘). In the adult brain, coexpression of Hbn and the nuclear marker RFP was also present in the medulla (Fig. 3E, white arrowhead) and the neighbouring central brain region (Fig. 3E, yellow arrowhead). In summary, the hbn^KOGAL4^ strain mimics the expression of Hbn in the brain during all developmental stages from the embryo to the adult. As already mentioned for the hbn^KO^ strain the hbn^KOGAL4^ strain shows also embryonic lethality. When we analysed homozygous embryos using HRP as a general marker, we detected the typical *hbn* phenotype [26] with an almost complete loss of the supraesophageal brain commissure (Fig. 3F, white arrowhead), misorganized protocerebral connectives (Fig. 3F, yellow arrowhead) and a reduction in the anterior part of the protocerebrum (Fig. 3F, red arrowhead) compared to the wild-type (Fig. 3G, white, yellow and red arrowheads). In the homozygous hbn^KOGAL4^ embryos, no Hbn expression was detected (data not shown).

### Generation of *hbn*-Gal4 strains

**Fig. 4:**
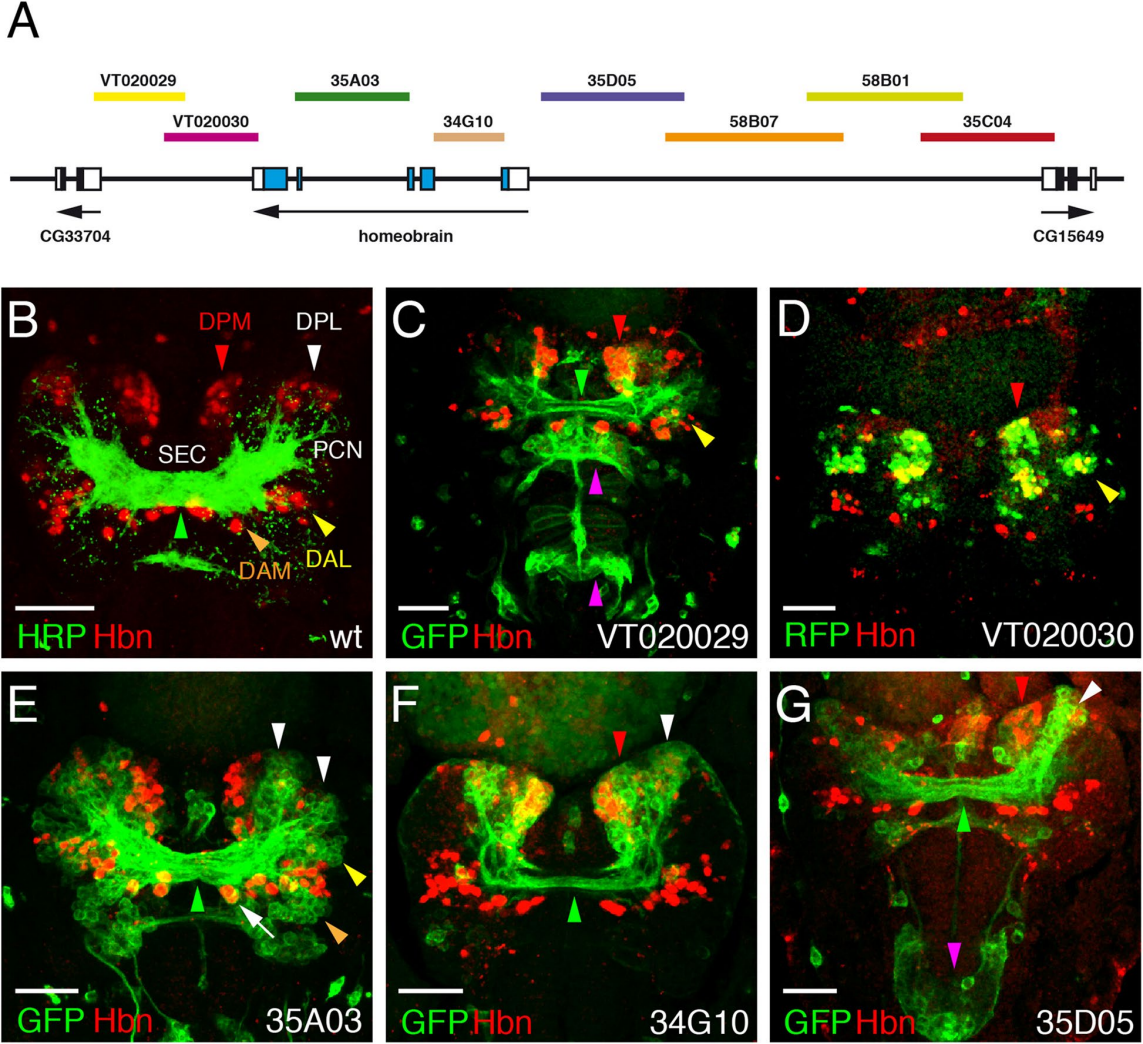
Expression of *hbn* enhancer-Gal4 strains in the embryo. **A** The genomic organization of the *hbn* locus is shown together with the locations of fragments from the upstream, intronic and downstream regions of the *hbn* locus used to test enhancer activities in the respective Gal4 strains. **B-G** Dorsal views of the anterior parts of stage 15 *Drosophila* embryos. The anterior ends of the embryos are pointing towards the bottom. An anti-Hbn antibody was used to visualize the nuclear Hbn expression pattern (red). Enhancer-Gal4-driven UAS-mCD8::GFP expression in the membrane (green, **C**, **E-G**) or UAS-H2B-mRFP1 expression in the nucleus (green, **D**) visualize the expression patterns generated by the various enhancers. In (**B**) HRP was used as a marker (green). The Gal4 strain numbers are indicated, and arrowheads indicate important regions showing coexpression of Hbn and the fluorescence marker (red arrowheads, DPM region; white arrowheads, DPL region; yellow arrowheads, DAL region; orange arrowheads, DAM region; green arrowheads, supraesophageal commissure; magenta arrowheads, clypeolabrum in (**G**) and additional staining domains in (**C**)) The white arrow in (**E**) indicates the Hbn cells in direct proximity to the commissure. Abbreviations: DAL, dorso-anterior lateral lineages; DAM, dorso-anterior medial lineages; DPL, dorso-posterior lateral lineages; DPM, dorso-posterior medial lineages; PCN, protocerebral connective; SEC, supraesophageal commissure. For symmetrical expression patterns, domain arrowheads are only shown on the right side. (Scale bars: **B**-**G**, 25 μm)

Due to the very complex expression pattern of Hbn in different developmental stages, we decided to perform a careful analysis of regulatory elements driving this expression pattern. Several years ago, a research project was initiated to analyse regulatory regions of 925 genes with a predicted function or expression in the adult brain using newly produced Gal4 strains [35]. In the course of this project, 5000 transgenic fly strains were planned and generated, including strains for *hbn*. Since we were generously provided information on which strains were planned for *hbn*, we were able to produce the constructs and transgenic strains in parallel on our own. For this experiment, we used the nomenclature of the Janelia Research Campus. The overlapping constructs 35C04, 58B01, 58B07 and 35D05 covered a region of 12 kb upstream of *hbn* up to the gene CG15649, and the constructs 34G10 and 35A03 included the two large introns of the *hbn* gene (Fig. 4A). These six constructs were cloned in the vector pBGUW and integrated in an attP docking site located at 65B2 on the third chromosome using PhiC31-mediated integration as reported by [35]. The established transgenic fly lines were balanced to be used in further experiments. In addition, two Gal4 strains VT020029 and VT020030, which covered 4.5 kb downstream of *hbn* up to the CG33704 gene, derived from the Vienna Tiles-Gal4 library, were included in the analysis (Fig. 4A). All strains were recombined with a UAS-mCD8::GFP, UAS-H2B-mRFP1 strain, which has both constructs also integrated on the third chromosome [66]. Either one or both of the markers are recombined with the Gal4 construct in the final strain and can be used directly for the analysis without further crossings.

### Analysis of *hbn* enhancer constructs

First the expression of all *hbn* Gal4 strains was analysed in stage 15 embryos focusing on the brain, where the most prominent expression of Hbn is detected. Our primary goal was to identify enhancers regulating the expression in the major Hbn domains and those that are involved in building the supraesophageal commissure, the brain structure that is missing in *hbn* mutants. We used mCD8::GFP as a marker for GFP expression in the membrane, except for strain VT020030, to observe the axonal projections. For comparison, the brain of a stage 15 embryo was stained with the general marker HRP to highlight the supraesophageal commissure (SEC) (Fig. 4B, green arrowhead) and the protocerebral connectives (PCN), as well as the major Hbn expression domains (Fig. 4B, red, white, yellow and orange arrowheads). These regions are most likely corresponding to the DPM (dorso-posterior medial), DPL (dorso-posterior lateral), DAL (dorso-anterior lateral) and DAM (dorso-anterior medial) lineages [67–69]. In strain VT020029 carrying a fragment from the 3′ region of *hbn* up to the neighbouring CG33704 gene, expression of the GFP marker is colocalized with Hbn in the DPM and DAL regions of the brain (Fig. 4C, red and yellow arrowheads), and is also detected in axons projecting into the supraesophageal brain commissure (Fig. 4C, green arrowhead). Additional staining of the GFP reporter was detectable in two other regions not related to Hbn expression (Fig. 4C, magenta arrowheads). The tested fragment in strain VT020030 overlapped with VT020029; here, coexpression of RFP and Hbn was again visible in the DPM and DAL regions (Fig. 4D, red and yellow arrowheads). In strain 35A03, which contains a fragment covering the largest intron of *hbn*, GFP expression was observed in the DPL region (Fig. 4E, white arrowheads), the DAL region (Fig. 4E, yellow arrowhead) and the DAM region (Fig. 4E, orange arrowheads) where Hbn was coexpressed. In this strain, the Hbn pioneer neurons closely associated with the commissure and forming pioneer tracts [26, 70] were positive. The very strong staining of the supraesophageal commissure in strain 35A03 (Fig. 4E, green arrowhead) indicated that this enhancer is responsible for driving the hbn expression in the majority of cells sending their axons to the midline and comprising the commissure. A fragment derived from the second largest intron in strain 34G10 induced coexpression in the DPM region (Fig. 4F, red arrowhead) and part of the DPL region (Fig. 4F, white arrowhead); and again some axons of the commissure expressed GFP (Fig. 4F, green arrowhead). Among the strains with fragments covering the upstream region of *hbn* up to the CG15649 gene, only strain 35D05 gave a strong expression. It bears a fragment from the region upstream of the transcription start site causing coexpression of the reporter with Hbn in the DPM region of the brain (Fig. 4G, red arrowhead) and part of the DPL region (Fig. 4G, white arrowhead). Axons projecting from that region to the midline were again part of the commissure (Fig. 4G, green arrowhead). In this strain, GFP expression was also observed in the clypeolabrum (Fig. 4G, magenta arrowhead), a region where Hbn was also expressed in earlier stages; therefore, GFP marker expression might persist, whereas Hbn expression was absent in stage 15 embryos. Strains 58B01 and 58B07 did not show any reporter expression in the embryo. In strain 35C04, only a few cells in the medial brain region showed expression of the RFP marker but no coexpression with Hbn (data not shown).

Our analysis identified several enhancers with defined expression patterns in different regions of the embryonic brain. Enhancers in four different regions of the *hbn* gene are responsible for the expression of Hbn in cells that project to the midline and are part of the supraesophageal brain commissure. This commissure consists of two dorsal commissural tracts and one ventral tract [70]. In the present study, the enhancer 35A03 was the most prominent enhancer, showing strong reporter gene expression in the commissure and therefore most likely of all three commissural tracts. In contrast, the other three enhancer regions (VT020029, 34G10 and 35D05) only showed expression in the dorsal commissural tracts. This observation was most evident when we compared the distance of the Hbn-expressing cells in close association with the commissure relative to the tracts marked by the GFP reporter. Here, in strains VT020029, 34G10 and 35D05, the Hbn cells were located at some distance to the tracts labelled by the reporter, whereas in strain 35A03 they were in direct proximity (highligthed by a white arrow in Fig. 4E).

**Fig. 5:**
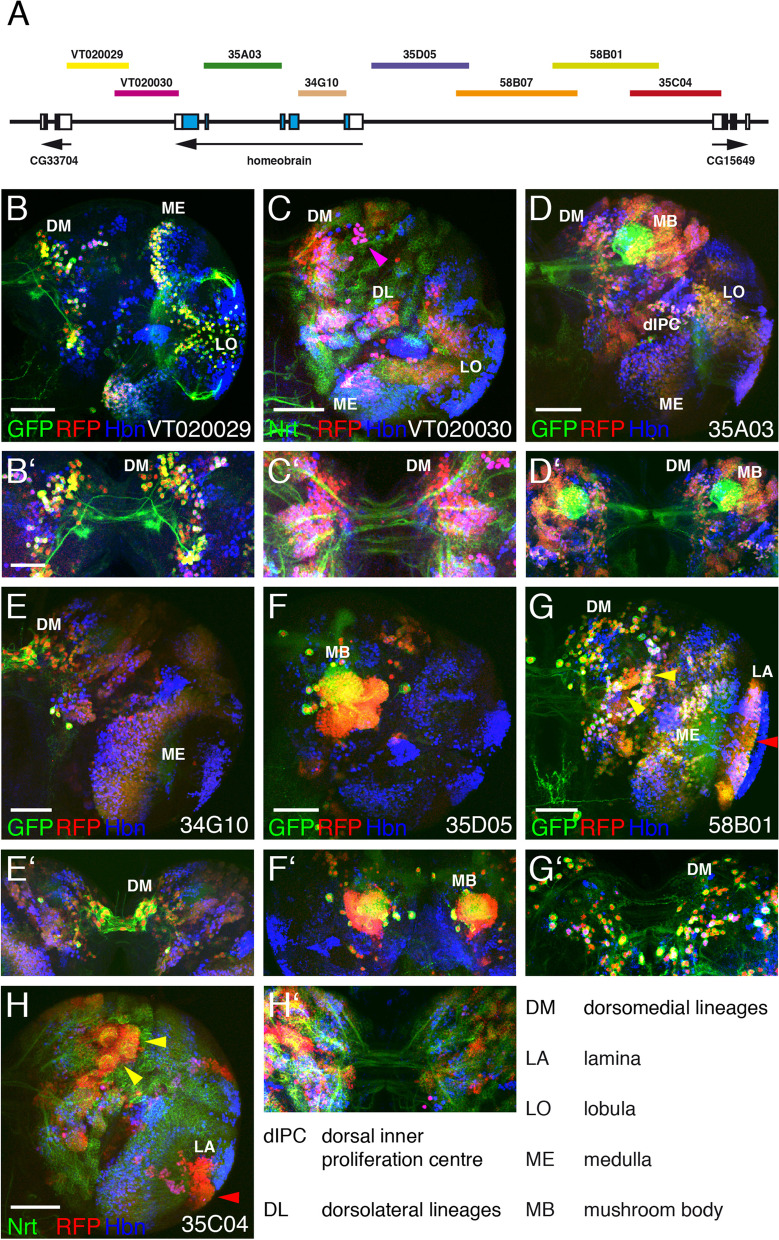
Expression of *hbn* enhancer-Gal4 strains in the larval brain. **A** The genomic organization of the *hbn* locus is shown together with the locations of fragments from the upstream, intronic and downstream regions of the *hbn* locus used to test enhancer activities in the respective Gal4 strains. **B-H** Views of right hemispheres of *Drosophila* L3 larval brains. **B‘-H‘ **Views of the central brain focusing on the commissure region. An anti-Hbn antibody was used to visualize the nuclear Hbn expression pattern in blue, and enhancer-Gal4-driven UAS-H2B-mRFP1 (red) and/or UAS- mCD8::GFP (green) expression visualize the patterns generated by the various enhancers. In (**C**) and (**H**) an anti-Nrt antibody (green) was additionally used. The Gal4 strain numbers are indicated, yellow arrowheads indicate important regions showing coexpression of Hbn and the fluorescence marker, magenta arrowheads coexpression in primary neurons in (**C**) and the lamina in (**G**), red arrowheads indicate regions where only the enhancer expression is visible. Abbreviations are indicated in the figure. (Scale bars: 50 μm, **B‘-H‘** as in **B‘**)

In the next step we analysed the expression of the same Gal4 strains in third instar larval brains. In strain VT020029, expression was detected in some cells in the DM region, which also extended axonal projections to the midline (Fig. 5B, B‘). In addition, expression was observed in the medulla (ME) and lobula (LO), but without coexpression of Hbn in the lobula (Fig. 5B). Strain VT020030, partly overlapping with VT020029, showed stronger expression in the DM lineages with projections to the midline and in the region of the DL lineages (Fig. 5C, C‘). A conspicuous large cell cluster next to the DM region most likely comprised primary neurons (Fig. 5C, magenta arrowhead). Additionally, expression in the medulla (ME) was detectable, but not in the lobula (LO) (Fig. 5C). Strain 35A03, which contains the fragment covering the largest intron of *hbn*, showed expression in the DM region with some projections to the midline, in the mushroom bodies (MB), the dorsal inner proliferation centre (dIPC), the medulla (ME) and the lobula (LO) (Fig. 5D, D‘). In strain 34G10 strong expression was observed in the DM region, with cells showing projections to the midline, and in the medulla (ME) (Fig. 5E, E‘). The region immediately upstream of the *hbn* transcription start site present in strain 35D05 induced expression in the mushroom bodies (MB); here, no midline projections were visible (Fig. 5F, F‘). Strain 58B01 showed expression in several type I lineages (Fig. 5G, yellow arrowheads), in the medulla (ME) and the lamina (LA) (Fig. 5F, red arrowhead), but Hbn coexpression was not observed in the lamina. Additionally, expression was observed in some cells in the DM region, and some midline projections were detectable (Fig. 5G, G‘). Strain 35C04, with the most upstream region of *hbn*, again showed marker expression in type I lineages (Fig. 5H, yellow arrowheads) and the lamina (LA) (Fig. 5H, red arrowhead), but no Hbn coexpression. Additionally, midline projections were detected in this strain (Fig. 5H‘). Since 58B01 and 35C04 overlap, the enhancer responsible for their common expression in the lamina might be located in this region, but this is definitively not an *hbn* enhancer, since Hbn is not expressed in the lamina. Instead, this enhancer might belong to the CG15649 gene or neighbouring genes further upstream of *hbn*. In summary, four strains showed expression in the DM region (VT020029, VT020030, 35A03 and 34G10), one in the inner proliferation centre (35A03), two in the mushroom bodies (35A03 and 35D05) and one in the lobula (35A03). Unexpectedly, medulla expression was observed in most strains.

**Fig. 6:**
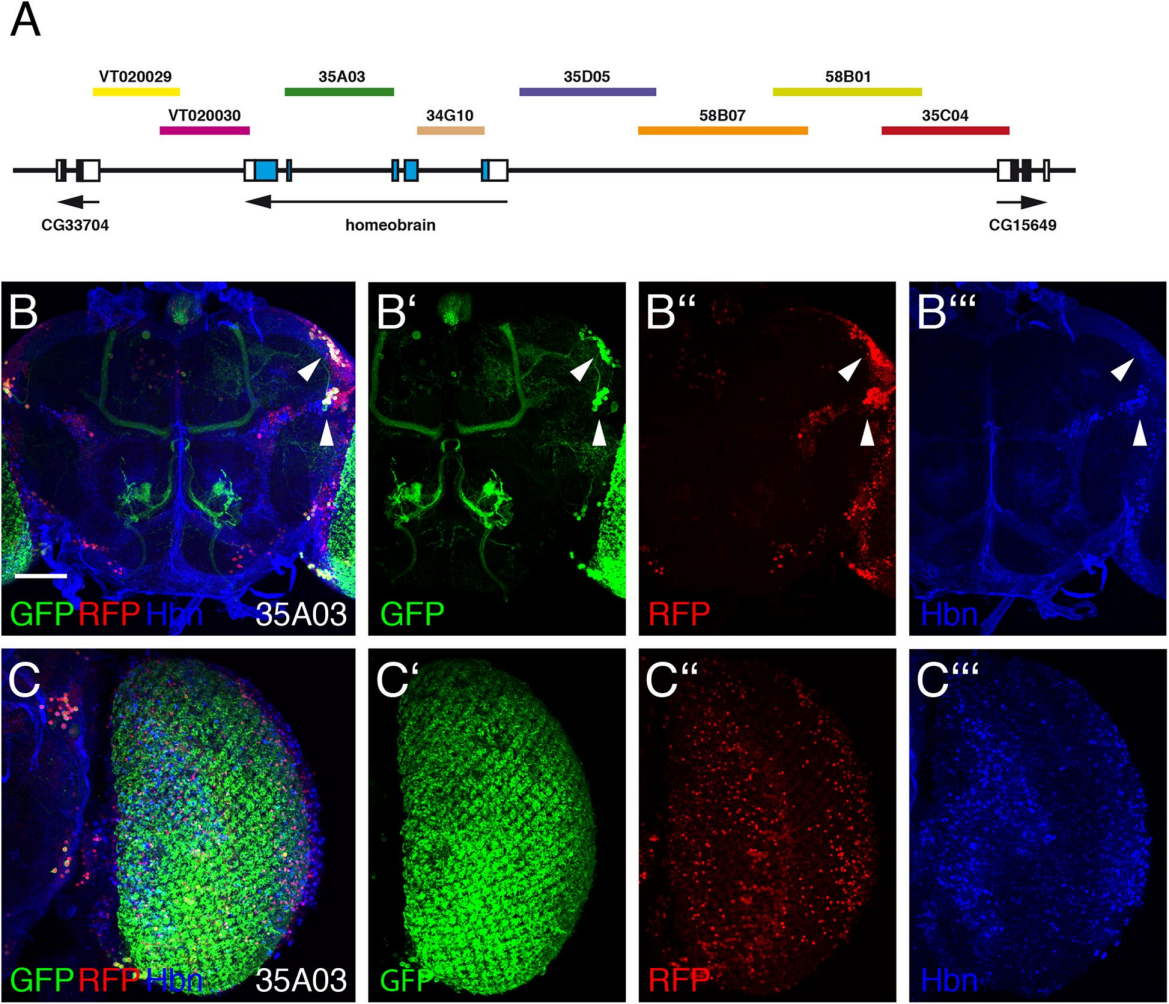
Expression of *hbn* enhancer-Gal4 strain 35A03 in the adult brain. **A** The genomic organization of the *hbn* locus is shown together with the locations of fragments from the upstream, intronic and downstream regions of the *hbn* locus used to test enhancer activities in the respective Gal4 strains. **B-B‘‘‘** Frontal view of an adult *Drosophila* brain of strain 35A03. **C-C‘‘‘** Adult optic lobe of strain 35A03. An anti-Hbn antibody was used to visualize the nuclear Hbn expression pattern in blue, and enhancer-Gal4-driven UAS-mCD8::GFP and UAS-H2B-mRFP1 expression visualized the patterns generated by the enhancer 35A03 in green (GFP) and red (RFP). White arrowheads indicate coexpression of Hbn and the markers in the central brain region. (Scale bar: 50 μm)

**Fig. 7:**
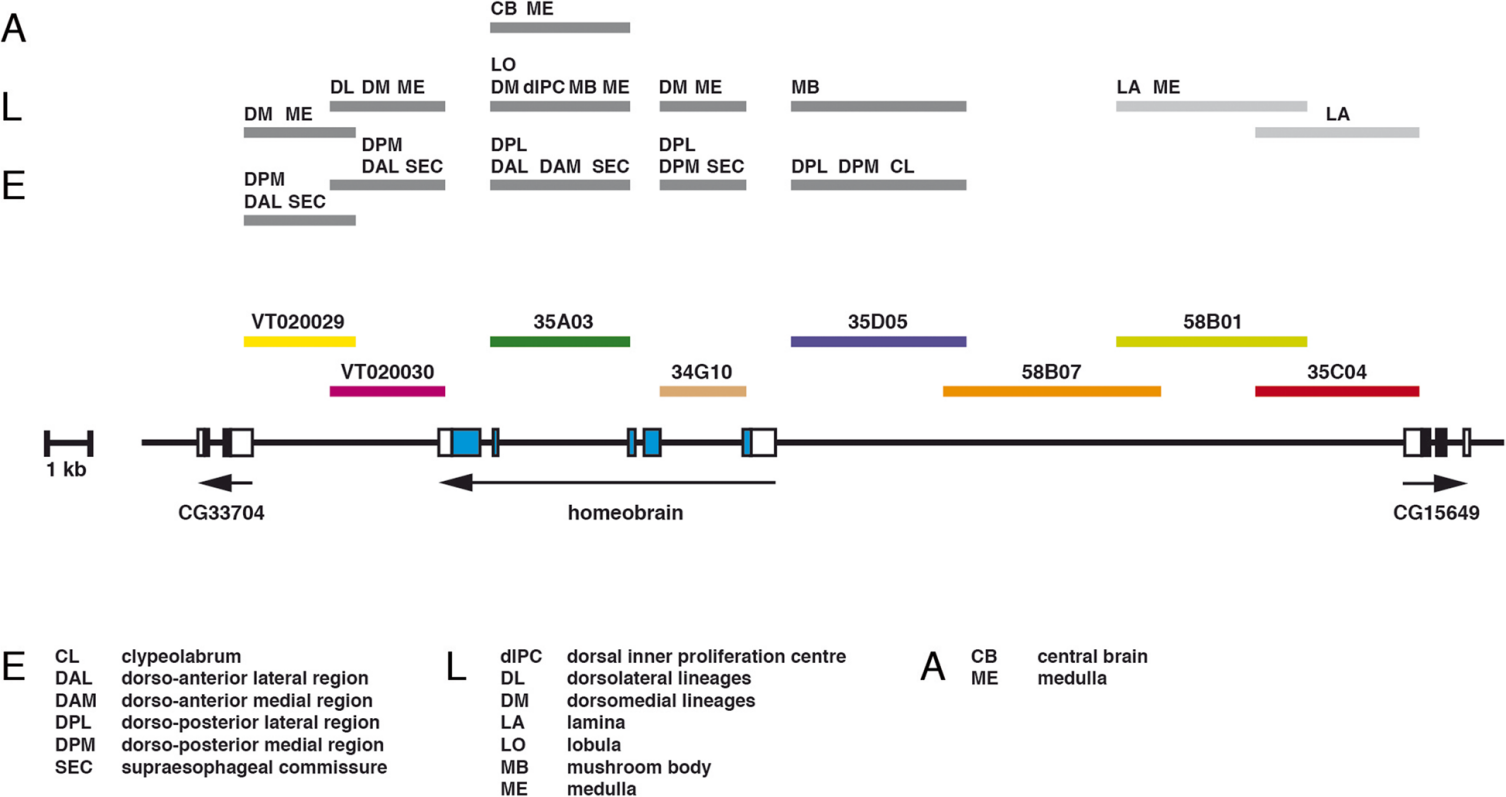
Location of putative enhancers of the *hbn* gene driving expression at different developmental stages. The genomic organization of the *hbn* locus is shown together with the locations of fragments from the upstream, intronic and downstream regions of the *hbn* locus used to test enhancer activities in the respective Gal4 strains. The locations of the different putative *hbn* enhancers are indicated above the genomic organization as dark gray boxes at different developmental stages in the embryonic brain (E), larval brain (L) and adult brain (A). Enhancers not related to *hbn* are indicated as light gray boxes. Abbreviations: CB, central brain; CL, clypeolabrum; DAL, dorso-anterior lateral region; DAM, dorso-anterior medial region; dIPC, dorsal inner proliferation centre; DL, dorsolateral lineages; DM, dorsomedial lineages; DPL, dorso-posterior lateral region; DPM, dorso-posterior medial region; LA, lamina; LO, lobula; MB, mushroom body; ME, medulla; SEC, supraesophageal commissure

In the adult brain, a clear coexpression with Hbn was observed in the central brain region (Fig. 6B-B‘‘‘, white arrowheads) and in the medulla (Fig. 6C-C‘‘‘) of strain 35A03. GFP reporter expression in the medulla was previously reported (Fly Light database, Janelia Research Campus). In all the other strains no colocalization of Hbn and the reporters was detectable. A schematic summary of the expression of all enhancers during development is shown in Fig. 7.

### Generation of *hbn* enhancer deletions by gene targeting

**Fig. 8:**
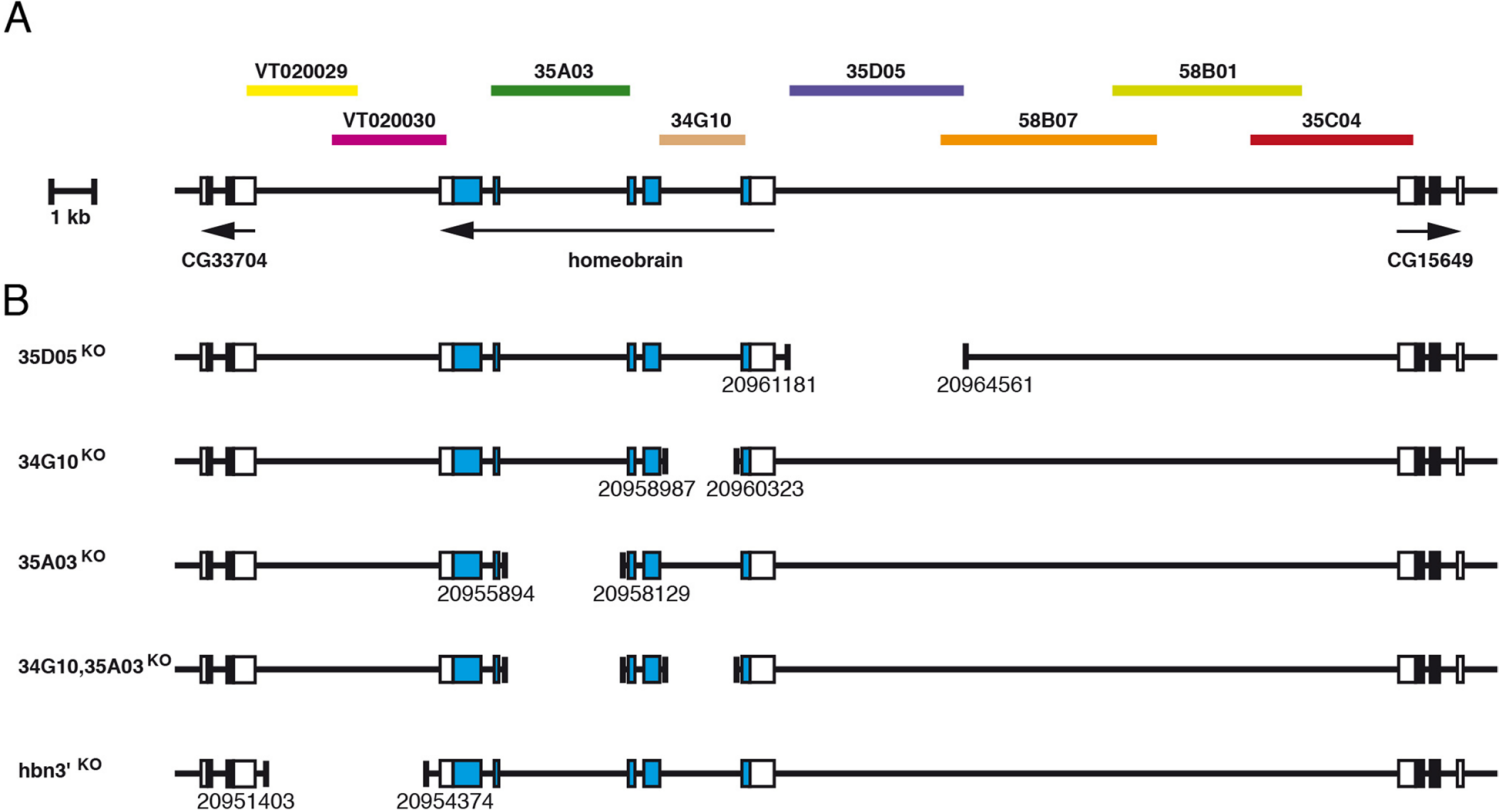
*Hbn* enhancer gene targeting strains. **A** The genomic organization of the *hbn* locus is shown together with the locations of fragments from the upstream, intronic and downstream regions of the *hbn* locus used to test enhancer activities in the respective Gal4 strains. **B** The individual enhancer deletion strains 35D05^KO^, 34G10^KO^, 35A03^KO^, 34G10,35A03^KO^ and hbn3‘^KO^ with respective deleted regions are indicated. Deletion breakpoint positions are indicated according to the sequences from Flybase (FB2021_04)

Our analysis of all putative enhancers from the *hbn* locus showed that four, 35D05, 34G10, 35A03 and the *hbn* 3′ enhancer region, account for the major expression pattern of Hbn and drive expression in specific areas in the embryonic brain, mushroom body progenitors and supraesophageal brain commissure. Later in development, they are responsible for expression in the type II lineages, the medulla and the lobula complex in the larval brain. We performed a functional analysis of these *hbn* enhancers by generating constructs for gene targeting experiments to delete individual enhancers via homologous recombination (Fig. 8). The 3’region contained two overlapping enhancer constructs (VT020029 and VT020030) that both showed activities. Here, we decided to create a single deletion of 3.0 kb, comprising the region downstream of *hbn* up to the neighbouring CG33704 gene which we called hbn3’^KO^. In order to avoid a general effect on both genes, we left 0.3 kb downstream of *hbn* and upstream of CG33704 intact. The other deletions were generated according to the same principle. The upstream deletion 35D05^KO^ covers 3.2 kb, yet contains 0.3 kb upstream of the *hbn* transcription start site. In the case of the two intron deletions, 0.2 kb neighbouring the donor and acceptor splice sites were left intact to avoid interfering with correct splicing, and perhaps a complete inactivation of *hbn*. The deletion of enhancer 34G10 (34G10^KO^) therefore has a length of 1.3 kb, and that of enhancer 35A03 (35A03^KO^) has a length of 2.2 kb. Since these two enhancer regions are responsible for predominant Hbn expression, we also decided to generate a deletion of both intronic regions at the same time (34G10,35A03^KO^). This was done by deleting the region 0.2 kb downstream of exon 1 up to the region 0.2 kb upstream of exon 4 and reintegrating the deleted exons 2 and 3 plus 0.2 kb upstream and downstream to again avoid a complete knockout of the *hbn* gene. For all these constructs, we used smaller homology arms ranging from 1.7 kb for the deletion of the 3′ enhancer and 2.5 to 2.7 kb homology arms for the other constructs. Similar to the *hbn* gene targeting construct, we PCR amplified the homology arms, cloned them in the pTV^cherry^ vector, produced transgenic flies and generated targeting flies through the appropriate fly crosses.

Homologous recombination occurred with a frequency ranging from 1 in about 1300 to 1 in about 6600, independent of the size of deletion introduced, or the size of the homology arms used (Table 1). In all cases, the *white* gene was removed, and the final strains were molecularly analysed using PCR and sequencing of the deletion breakpoints. The five strains were then balanced and analysed. Whereas 35D05^KO^, 34G10^KO^, 35A03^KO^ and 34G10,35A03^KO^ fly strains are viable, hbn3’^KO^ animals die during larval stages. The donor strain used for the hbn3’^KO^ targeting already showed larval lethality due to remaining P-element sequences in this strain. Since the insertion of the donor construct was similar to *hbn* also on the second chromosome, these remaining P-element sequences were not lost over time, perhaps explaining the hbn3’^KO^ larval lethality.

**Table 1:** Statistics concerning the generation of the *hbn* enhancer gene targeting strains

Strain	# of flies screened	red-eyed flies	positive results	size of deletion	size of HR arms
hbn^KO^	18,000	30	1/600	0.17 kb	4.0 kb
35D05^KO^	27,414	7	1/3916	3.2 kb	2.5–2.7 kb
34G10^KO^	56,087	40	1/1402	1.3 kb	2.5–2.7 kb
35A03^KO^	32,892	5	1/6596	2.2 kb	2.5–2.7 kb
hbn3’^KO^	47,814	16	1/1298	3.0 kb	1.7 kb
34G10,35A03^KO^	61,701	11	1/5609	4.3 kb	2.5–2.7 kb

### Functional analysis of *hbn* enhancer deletion strains

**Fig. 9:**
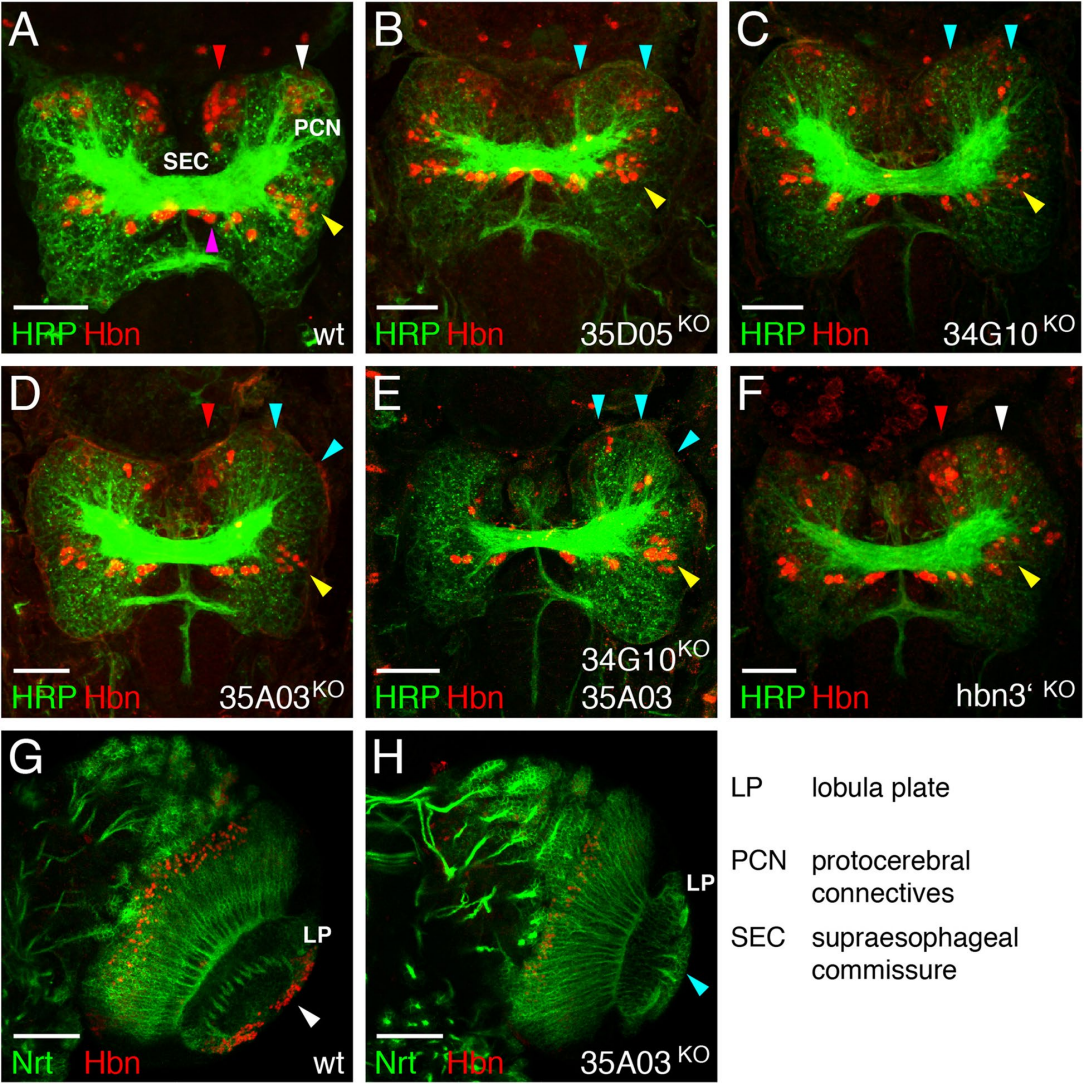
Analysis of *hbn* enhancer gene targeting strains. **A-F** Laser confocal images of stage 16 embryonic brains from the different *hbn* enhancer targeting strains compared to a wild-type brain stained with antibodies against HRP (green) and Hbn (red). In the wild-type brain, the supraesophageal commissure (SEC) and the protocerebral connectives (PCN) are the most prominent structures visible (**A**), but the sizes of these structures are reduced in all the *hbn* enhancer gene targeting strains (**B-F**). **A** Hbn is expressed in the medial region (red arrowhead), central region (white arrowhead), lateral region (yellow arrowhead) and in close association with the commissure (magenta arrowhead). **B** In strain 35D05^KO^, Hbn expression is reduced in the medial and central regions (blue arrowheads). **C** Strain 34G10^KO^ shows reduced Hbn expression again in the medial and central regions (blue arrowheads). **D** In strain 35A03^KO^, Hbn expression in the central and lateral regions is substantially reduced (blue arrowheads). **E** Strain 34G10,35A03^KO^ shows the most dramatic phenotype with the most substantial decreases in the sizes of the commissure and PCNs. Hbn expression is almost completely absent in the medial and central regions (blue arrowheads), and the brain size is smaller. **F** Strain hbn3’^KO^ shows a slight reduction in Hbn expression in the medial, central and lateral regions. **G**, **H** Laser confocal images of right hemispheres from third instar larval brains stained with Nrt (green) and Hbn (red) antibodies. **G** In the wild-type optic lobe, Hbn is expressed in the lobula plate (LP) (white arrowhead). **H** In the 35A03^KO^ strain, Hbn expression in the lobula plate is not observed (blue arrowhead). Abbreviations are indicated in the figure. (Scale bars: **A-F**, 25 μm; **G**, **H**, 50 μm)

Puzzled by the viability of the enhancer deletion strains we wondered, whether *hbn* expression in the brain is affected as predicted from the respective enhancer activity outlined above. Hence, we performed a detailed analysis of the *hbn* enhancer deletion strains, by combining Hbn staining in embryonic and larval brains with several general markers. We expected to observe a loss of Hbn expression in distinct areas and perhaps alterations in specific brain structures. For staining of stage 15 embryonic brains, HRP was used as a general marker together with Hbn. We combined confocal stacks from the middle part of the brain at the level of the supraesophageal commissure showing adjacent domains of Hbn expression (Fig. 9A-F). In a wild-type brain the supraesophageal brain commissure (SEC) and the protocerebral connectives (PNC) were the most prominent brain structures visible (Fig. 9A). Hbn expression in the brain was visible in a dorsomedial domain (red arrowhead), a central domain (white arrowhead), a lateral region (yellow arrowhead) and closely associated with the commissure (magenta arrowhead). A common feature of all enhancer gene targeting strains was that the supraesophageal commissure was thinner than the wild-type structure, and also the protocerebral connectives appeared to be slightly reduced (Fig. 9B-F), phenotypes typical of *hbn* mutants [26]. In strain 35D05^KO^ the Hbn expression was reduced in the dorsomedial and central regions (Fig. 9B, blue arrowheads), where the 35D05 enhancer drives the expression of Hbn. Moreover, the brain hemispheres appeared smaller. A similar effect was observed in strain 34G10^KO^; here again, the expression in the dorsomedial and central brain regions was reduced (Fig. 9C, blue arrowheads). If the enhancer 35A03, which drives expression in the central and lateral domains, was deleted in strain 35A03^KO^, almost no Hbn expression was detected in these areas (Fig. 9D, blue arrowheads). The two *hbn* enhancers 34G10 and 35A03 are the two enhancers responsible for expression in many areas also during later developmental stages, and were, hence deleted simultaneously in strain 34G10,35A03^KO^. An almost complete disappearance of Hbn expression was observed in the dorsomedial, central and lateral brain regions (Fig. 9E, blue arrowheads) and a reduction of the supraesophageal commissure was detected. The hbn3’^KO^ strain showed the weakest effect; here, Hbn expression was only slightly reduced (Fig. 9F). In the larval stage, the enhancer 35A03 was the only *hbn* enhancer driving expression in the lobula plate (Fig. 9G, white arrowhead) and this expression was lost in strain 35A03^KO^ (Fig. 9H, blue arrowhead).

## Discussion

As shown in our previous studies, Hbn is expressed in specific regions of the protocerebrum in the embryo, including neuroblasts, GMCs and neurons and in the mushroom body progenitor cells [26]. In this paper, we continued this analysis of Hbn expression in postembryonic stages. We showed, that Hbn is expressed in all DM and DL lineages of the larval brain. The neuroblasts that generate these lineages are already present in the embryo in three clusters; an anterior dorsomedial (ADM) cluster of three neuroblasts, a posterior dorsomedial (PDM) cluster of three neuroblasts and a dorsolateral (DL) cluster of two neuroblasts [9, 10]. The ADM cluster generates the DM1–3 lineages, the PDM cluster produces the DM4–6 lineages and the DL cluster generates the DL lineages. Embryonic type II neuroblasts express a series of some well-known transcription factors, including Hbn, DRx and Otp [17]. Hbn and DRx are only expressed in the PDM and DL clusters which are missing when both genes are deleted, whereas the ADM cluster is still present [17]. Even if Hbn is not expressed in the ADM cluster, it is expressed in all type II lineages in the larval brain, including INPs, GMCs and neurons in part of the lineages. Perhaps Hbn is expressed in the neuroblasts in the DM1–3 lineages during early larval stages, or some redundancy exists such that only the inactivation of several factors at one time produces well-defined phenotypes. Since a time series of transcription factors (Dichaete, Grainyhead and Eyeless) is present in the DM lineages in the larval brain [29], Hbn might also be one of these factors that is expressed in the INPs and cells derived from them. Our experiments show that Hbn is expressed more distally in the DM lineages than Eyeless, with overlap observed in a few cells. A recent analysis very nicely showed that Hbn is indeed part of this time series in the DM lineages and that Eyeless binds the *hbn* locus and could repress Hbn expression [71]. The sequential activation of these factors in INPs generates different neurons and glia over time and thereby increases neural diversity in the adult brain [29]. In the optic lobe, Hbn is expressed in the medulla, where it is definitively part of a time series of transcription factors, as shown recently [33]. Here, Hbn expression almost completely overlapped with Eyeless expression in neuroblasts just before Sloppy paired 1 expression occurs. In addition, Hbn is required for Traffic Jam [72] and Orthodenticle [73] transcription factor expression in neurons. Therefore, Hbn has a dual role in the optic lobe, and it is necessary for the time series to progress by activating Sloppy paired 1, but also regulates factors necessary for the generation of neuronal progeny [33]. Additional expression is detected in the lobula plate; studies to determine how this expression pattern is related to other known factors expressed in that structure will be interesting [74].

We have analysed eight enhancer candidate fragments and identified five fragments resulting in marker expression patterns that were clearly assigned to the Hbn expression. The enhancer fragments are located in the upstream, intronic and downstream regions of *hbn*. These results are consistent with an earlier study showing that 46% of 7705 tested candidate fragments are active in the embryo and are located upstream (30%), downstream (22%) or in introns (36%) and that on average, a *Drosophila* gene contains four enhancers [75]. Since 12% of all enhancers are located further away, additional *hbn* embryonic enhancers might exist. The enhancer elements we identified, however, reflect the complete Hbn expression pattern during development in general, rendering this hypothesis rather unlikely. Of course, we cannot exclude the presence of redundant enhancers, called shadow enhancers known to provide robustness to regulatory networks [76, 77]. In fact, our study showed that expression in a specific region was regulated by several enhancers, such as the DM lineages and the medulla of the larval brain. Here, one enhancer might be a shadow enhancer, as it was shown for the *Drosophila snail* gene [76] and the gap genes *knirps* and *Krüppel* [78]. More systematic analyses of 1000 predicted shadow enhancers revealed that 64% of the loci examined had shadow enhancers, and 70% of these loci had even more than one shadow enhancer [76]. Thus, shadow enhancers are also very likely present among the identified *hbn* enhancers. The next level in the analysis of these enhancers might be to examine sequence conservation within other *Drosophila* species, to identify putative transcription factor binding sites and to identify sequence conservation among enhancers regulating expression in the same structure.

Using gene targeting technology and the vector pTV^cherry^ [65], we established 6 different donor constructs to generate respective targeting strains. One was used for deleting the N-terminal part of the *hbn* coding region, thereby generating an enhancer trap Gal4 line. The others allowed to establish five different strains with deletions of defined enhancer regions alone or in combination. The efficiency of the targeting mainly depends on three parameters; the length of the homology arms, the size of the region which has to be deleted and the chromosomal integration of the donor targeting construct used for the targeting via homologous recombination. The initial use of the vector pTV^cherry^ by [65] for six constructs resulted in a targeting efficiency of 1/1000 up to 1/3000 for five constructs and 1/8000 for the other. In these experiments, homology arms of 3–5 kb in length were used [65]. We generated our first targeting constructs with the pTV^cherry^ vector for the genes *hbn* (this study) and *DRx* [55] with 4 kb homology arms and obtained comparable efficiency (1/600–1/700), which was even better than the previously published efficiency [65], potentially because the deleted regions (0.17 kb and 0.39 kb) are rather small. For four of the enhancer deletion constructs we reduced the size of the homology arms to 2.5–2.7 kb for easier amplification and cloning. Since the deleted regions were also larger, the efficiency decreased to 1/1400–1/6600 for the enhancer deletion constructs. For the 3′ enhancer deletion constructs we reduced the homology arm length to 1.7 kb but still obtained good efficiency (1/1300). The variations might also depend on the initial integration sites of the constructs, since we observed differences in the efficiency of the 34G10 enhancer deletion ranging from 1/910–1/3300, depending on the chromosomal integration site. We did not reduce the homology arm length any further, but we propose that a homology arm length ranging from 1.7 kb to 2.5 kb is a good choice for deletions up to 3.5 kb. The advantage of an analysis using these enhancer deletions compared to downregulation with RNAi is that the deletions represent new *hbn* alleles with a well-defined and reproducible phenotype, whereas the expression of RNAi constructs for a gene using specific enhancer constructs and the UAS/Gal4 system might result in a temporal delay of the downregulation process and/or prolonged downregulation. Additionally, the downregulation of a gene using RNAi is never as effective as a complete inactivation, which is most likely also the case for the inactivation of an enhancer.

The effects of enhancer deletions are most obvious in the embryonic brain; here, deletions of individual enhancers lead to a loss of Hbn expression in the domains where the enhancers are normally active. Of course, a loss of expression in a specific area might not lead to a loss of all the cells where Hbn is expressed, but at least some cells might be lost since we detected a reduction in size of the supraesophageal commissure, alterations in the protocerebral connectives and also a slight reduction in size of the protocerebrum in all strains, phenotypes typical for *hbn* mutants [26]. In particular, the generation of the supraesophageal commissure is interesting; here, protrusions of both brain hemispheres extend towards the midline and form an interhemispheric cell bridge [79]. Then, fibre tract founder clusters and their axons form a system of pioneer tracts in temporal order starting with clusters P2l and P2m [70]. The generation of these initial tracts depends on the enhancer 35A03, but other tracts are formed even in the absence of this enhancer, arguing for an independent mechanism underlying the formation of all tracts and not a successive tract formation where the construction of pioneer tracts is necessary for the following tracts to form. The simultaneous deletion of enhancers 35A03 and 34G10 similarly exerted an additive effect on commissure formation, protocerebral connective formation and brain size. In the larval brain, the enhancer 35A03 regulates the Hbn expression in the lobula, and a deletion of this enhancer leads to a complete loss of Hbn expression in the lobula. Concerning the medulla expression, the effects of enhancer deletions are more difficult to identify, since Hbn is expressed in different periods and different cell types in the medulla. Due to the regulation of other factors in the medulla by Hbn and its regulation by other factors [33], the effects might only be identified using these recently identified interacting factors as markers.

The analysis of gene functions by creating deletions has been performed for many years, especially using the gene targeting technologies in mice [80]. This technology was first used to inactivate genes and later also for the functional analysis of enhancers such as a mouse HoxD enhancer [81] or two enhancers affecting the mouse genes H19 and Igf2 [82]. Later, this technology was also used in *Drosophila* [40–42] for comparable studies of genes and enhancers. In most of these enhancer studies, single enhancers were analysed at a time, but three enhancers of the *rhomboid* gene with a length between 0.3 kb and 1.0 kb were analysed alone or in combination using the CRISPR/Cas9 system in a study to uncouple neurogenic gene networks [83], as well as in the analysis of the *brinker* gene where two enhancers of 1.0 kb and 1.5 kb were deleted [84]. In our study, we decided to use gene targeting for the precise deletion of larger enhancer regions. To our knowledge, this analysis and our recently published analyses of Erm [85] and DRx [55] are the first in which several larger enhancer regions were deleted alone and in one combination in *Drosophila*. In mice, a larger enhancer deletion analysis was made by genome editing of 23 enhancers from seven loci required for limb development [86]. None of the 10 individual enhancer deletions resulted in discernible phenotypes, in contrast to deletions of enhancer pairs, suggesting the functional redundancy of enhancers [86]. This redundancy might also apply to some processes regulated by the enhancers we have characterized. Our analysis of the *hbn* enhancer deletion strains suggested reduction of Hbn expression in specific areas in the embryonic brain, affecting the size of supraesophageal commissure and the protocerebral connectives. Interestingly none of the enhancer deletions resulted in lethality as one would have expected from the *hbn* mutant phenotype. Since animals of the weaker *hbn*^4028^ allele survive in 20% of the cases up to the first instar larval stage and show a very thin commissure [26], one could assume that through the action of shadow enhancers a reduction of cells in the protocerebrum and a thinner commissure in our enhancer deletions is compensated up to a point which allows survival up to the adult. Another possibiltiy might be the presence of not yet identified additional *hbn* shadow enhancers.

## Conclusions

We showed that the *Drosophila* homeodomain transcription factor Hbn has a dynamic expression pattern during development up to the adult stage, emphasizing its role for brain development. The full complexity of the *hbn* expression pattern was recapitulated by the newly generated *hbn*-Gal4 strain. The *hbn* regulatory region was subdivided into several well-defined enhancers, located upstream, downstream as well as within two large introns of the *hbn* gene. Several *hbn* enhancer deletion strains established by gene targeting enabled us to assign phenotypic alterations typical for *hbn* mutations to specific *hbn* enhancer regions, allowing further exploration of these *hbn* enhancers in the future.

## Methods

### Fly strains

The following fly strains were used: yw^67c23^; UAS-mCDC8-GFP, UAS-H2B-mRFP1 [66], ubiquitin-Gal4[3xP3-GFP] [65].

The following stocks were obtained from the Bloomington Drosophila Stock Center:

y [1] w[67c23]; sna[Sco]/CyO, P{w[+mC] = Crew}DH1 (BL 1092);

y [1] w[*]; Pin[Yt]/CyO; P{w[+mC] = UAS-mCD8::GFP.L}LL6 (BL 5130);

y [1] w[1118]; P{ry[+t7.2] = 70FLP}23 P{v[+t1.8] = 70I-SceI}4A/TM3, Sb [1] Ser [1] (BL 6935);

w[1118]; Df(2R)Exel7166/CyO (BL 7998);

y [1] w[1118]; PBac{y[+]-attP-3B}VK00033 (BL 9750);

y [1] w[*]/Dp(2;Y)G, P{w[+mC] = hs-hid}Y; P{ry[+t7.2] = 70FLP}23 P{v[+t1.8] = 70I-SceI}4A/TM3, P{w[+mC] = hs-hid}14, Sb [1] (BL 25679).

y [1] w[*] P{y[+t7.7] = nos-phiC31\int.NLS}X; sna[Sco]/CyO (BL 34770);

w[1118]; P{y[+t7.7] w[+mC] = GMR34G10-GAL4}attP2 (BL 49802);

w[1118]; P{y[+t7.7] w[+mC] = GMR35A03-GAL4}attP2 (BL 49812).

The following strains were obtained from the Vienna Drosophila Resource Center:

P{VT020029-GAL4}attP2;

P{VT020030-GAL4}attP2.

### Generation of an *hbn* gene targeting construct

An *hbn* donor gene targeting construct was made in the vector pTV^cherry^ according to [65]. The two 4.0 kb homology arms were amplified using Pfu DNA Polymerase (New England Biolabs) and BACR10P11 DNA [87]. Primers hbnGT1 (5′-TATAGCGGCCGCGCGGTTGCTAGCCAACC-3′) and hbnGT2 (5′-TATACCGCGGGAGCAACTCGCGATCCGTACG-3′) were used for homology arm 1, and hbnGT3 (5′-TATAACTAGTGTTTTAGTTTAACAAATATAAACTGGGG-3′) and hbnGT4 (5′-TATAGGCGCGCCGGCGACGAATTTTCAGTCCGAG-3′) were used for homology arm 2. All primers contained unique restriction enzyme recognition sites, which were added to their ends (underlined), enabling later cloning in the final vector. After the addition of 3′ adenine overhangs to two PCR products, they were subcloned into the vector pCR-XL-TOPO (ThermoFisher Scientific, Waltham, Massachusetts, USA) and checked by sequencing. From the correct clones, homology arms were excised with the relevant restriction enzymes and finally cloned into the vector pTV^cherry^ [65]. P-element-mediated transformation of some constructs into *w*^*1118*^ flies was performed by Bestgene (Chino Hills, California, USA). Transformants were balanced, and transformants with integration on the third chromosome were used to generate the final targeting strain. Transformants were crossed with *hs-Flp, hs-SceI* flies (BL 6935), and resulting larvae were heat shocked at 48 h and 72 h after egg laying for 1 h at 37 °C. Two hundred adult female flies with mottled red eyes were crossed with *ubiquitin-Gal4[3xP3-GFP]* males, and the progeny were screened for the presence of red-eyed flies. The transgene *ubiquitin-Gal4[3xP3-GFP]* was removed by selection against GFP expression and the resulting targeting flies were balanced over CyO and molecularly analysed for the correct integration event. To verify this finding, we performed PCR reactions with primers within the cassette introduced by the recombination events and primers located outside of the homology arms (hbnGT1B (5′-CCACTACGTTTGGATGGGGCTG-3′), mCherryrev2 (5′-CCTCGTCGTCGTTCAGGTTG-3′) for the upstream region and pTVGal4–1 (5′-CGTTTTTATTGTCAGGGAGTGAGTTTGC-3), hbnGT4A (5′-CGCTCGTCGGACAAAAGGGTG-3′) for the downstream region). From one of these strains, hbn^KO^, removal of the *white* gene was performed by crossing of the hbn-targeting flies to a strain expressing Cre recombinase (BL 1092) and selecting for and balancing of white eyed flies among the cross offspring. For the reintegration of Gal4 in the *hbn* locus the vector RIV^Gal4^ was used [65]. Hbn-targeting flies were crossed with PhiC31-expressing flies (BL 34770) and embryos of that cross injected with RIV^Gal4^ DNA. Red-eyed transformant flies were selected and the *white* marker was again removed using the loxP sites to generate the strain hbn^*KOGal4*^.

### Generation of *hbn* Gal4 constructs

Amplifications of the *hbn* upstream and intronic regions were performed using BACR10P11 DNA [87]. For polymerase chain reactions Taq Polymerase from ThermoFisher Scientific (Waltham, Massachusetts, USA) was used according to supplier’s instructions. The primers used were 35C04F (5′-TTCCCATTTCGCCGTTTGCCAGGCTTAATCC-3′) and 35C04R (5′-GTTATTTGATCTGATGTTTTCGAACCGCATCGTG-3′), 58B01F (5′-TGAAAACATCGCAATCAGGGGGCTCATG-3′) and 58B01R (5′-TTTTCTTTGTTCTGCATTTAGCTGGGCCGC-3′), 58B07F (5′-TTGCAATTGCACATAAACTGTGTTGATATTGGGTCC-3′) and 58B07R (5′-AGTTTTAACTTGCCGTGGAGGGTGGC-3′), 35D05F (5′-ACATTTTCCCAGGCAGCAACGGCGC-3′) and 35D05R (5′-TTTTGGCTTAGGGGTGTTGGCCTCCTCC-3′), 34G10F (5′-AATCAGCACCAGATCAAGCGCAGTGGTAG-3′) and 34G10R (5′-AAATGATGGATGTGATGCGGATGGCCG-3′) and 35A03F (5′-TTTTCACAAGGGAGGATCTGGCCATGCG-3′) and 35A03R (5′-ATTTGATGGGTGGATTCGTGAGATGGGG-3′). All PCR products were first subcloned into the Gateway vector pCR8/GW/TOPO (ThermoFisher Scientific, Waltham, Massachusetts, USA). DNA from these clones was transferred into the destination vector pBGUW (Pfeiffer et al., 2008) by a recombination reaction using a Gateway Clonase Enzyme Mix (ThermoFisher Scientific, Waltham, Massachusetts, USA). PhiC31-mediated transformation into flies with an attP docking site on the third chromosome at 65B2 (BL 9750) was performed by BestGene (Chino Hills, California, USA).

### Generation of *hbn* enhancer deletions by gene targeting

*Hbn* donor constructs for the deletion of enhancer regions were generated in the same way as was described for the *hbn* gene targeting construct using BACR10P11 DNA [87]. In all cases, homology arms of 2.5 kb to 2.7 kb were PCR-amplified using GT1 and GT2 primers for homology arm 1 and GT3 and GT4 primers for homology arm 2, except for the hbn3GT construct, here homology arms of 1.7 kb were PCR-amplified. The following primers were used: 35D05GT1 (5′-GCGGCCGCTATATGGCTTAACTACATAGCATTGTAACTCG-3′), 35D05GT2 (5′-GGTACCATGCGGCAGCAAATGAGCAACTGC-3′), 35D05GT3 (5′-ATCAGTCCAAAAGCTACCTGCAACCCAAACTCACAC-3′) and 35D05GT4 (5′-GGCGCGCCTGAGATCATGGCCATTGTTCAGACTGG-3′) for the construct 35D05GT; 34G10GT1 (5′-GAATTCAAGGGCATGTCCTGGGCTG-3′), 34G10GT2 (5′-GCGGCCGCTAACGCAGATCGCCGACG-3′), 34G10GT3 (5′-ACTAGTGAGGATATAACTACTTCAGCCACAATTG-3′) and 34G10GT4 (5′-GGCGCGCCGACGAATTTTCAGTCCGAGCC-3′) for the construct 34G10GT; 35A03GT1 (5′-GAATTCGTACGGATCGCGAGTTGC-3′), 35A03GT2 (5′-GGTACCATTAGCGGACACTGCGATGGC-3′), 35A03GT3 (5′-ACTAGTCCTCTCTTCGGTAAATGATATATCAG-3′) and 35A03GT4 (5′-GGCGCGCCAGGACCTTCCATTCG − 3′) for the construct 35A03GT; hbn3GT1 (5′-GCGGCCGCAAGGAGAGCCGAGTGCTGCTG-3′), hbn3GT2 (5′-GGTACCTAGCTGTTAACCAGAGCGCATAGTCG-3′), hbn3GT3 (5′-ACTAGTGGTAGATCGGTGTATGTATGTATGTTGTGG-3′) and hbn3GT4 (5′-GGCGCGCCGTATACAGGTTGTATACATAAGTCAGAAAAAGGC-3′) for the construct hbn3GT. To confirm that the deletions conformed to the prediction, we PCR-amplified the deletion breakpoints and the PCR products were sequenced by Starseq (Mainz, Germany). The following cloning steps were done in a similar way as for the *hbn* gene targeting construct. For the 34G10,35A03GT construct, we first combined the upstream homology arm 1 from construct 34G10GT and the downstream homology arm 2 from construct 35A03GT and generated a strain that had a deletion of the regions 34G10 and 35A03 including the *hbn* exons 2 and 3 and the small intron in between. These two exons and the small intron in between were afterwards reintegrated using the vector RIV^white^ [65]. Red-eyed transformant flies were selected and the *white* marker was again removed using the loxP sites to generate the final strain.

### Immunostaining

Embryos were collected, dechorionated with 50% bleach for 2 min, washed with 0.1% NaCl /0.1% Triton X-100 and fixed for 12 min in 3.7% formaldehyde in PEM (100 mM PIPES, 1 mM EGTA, 1 mM MgCl_2_) and heptane. After removal of both phases, embryos were devitelinized in equal volumes of heptane and methanol by 2 min of vigorous shaking and washed three times with methanol. The 3rd instar larvae and adult brains were dissected in 1x phosphate buffered saline (PBS), fixed for 60 min in 2% paraformaldehyde in PBL and washed three times with 1x PBS containing 0.2% Triton X-100 (PBX) and then incubated for 3 × 5 min in methanol. Fixed embryos or larval brains were washed 3 × 5 min and 6 × 30 min in PBX and blocked for 30 min in 5% normal horse serum and 10% PBX in PBS. Incubations with primary antibodies were performed overnight at 4 °C. Samples were washed 3 × 5 min and 6 × 30 min in PBX and blocked for 30 min in 5% normal horse serum and 10% PBX in PBS. After an overnight incubation with secondary antibodies at 4 °C embryos or larvae were washed 3 × 5 min and 6 × 30 min in PBX and mounted in Vectashield (Vector Laboratories). Adult brains were treated the same as larval brains but were incubated with the appropriate antibody two nights each. Images were obtained using a Leica TCS SP5 microscope (Leica, Wetzlar, Germany) or a ZEISS LSM 710 microscope (Carl Zeiss AG, Oberkochen, Germany) for laser confocal microscopy. Usually z-stacks of 1 μm were generated and several stacks combined to show the relevant structure or expression domain completely. Images were processed using FIJI and ImageJ (NIH. Md., USA), Adobe Photoshop and Adobe Illustrator (Adobe Systems, San Jose, CA, USA).

Primary antibodies used were guinea-pig anti-Hbn antibody (1:1000) [26], rabbit anti-DRx antibody (1:1000) [24], goat FITC-conjugated anti-HRP antibody (1:100) (ICN Biomedical/ Cappel), guinea pig anti-Dpn antibody (1:500) (a gift from Jürgen Knoblich), rabbit anti-Ey antibody (1:1000) (Uwe Walldorf), rabbit anti-Toy antibody (1.200) (Uwe Walldorf); rabbit anti-Elav antibody (1:30), mouse anti-Repo antibody (1:10), mouse anti-Pros antibody (1:10), mouse anti-Brp (nc82) (1:25) and mouse anti-Nrt (BP106) antibody (1:25) were obtained from the Developmental Studies Hybridoma Bank, Iowa. Secondary antibodies were goat anti-mouse, goat anti-rabbit and goat anti-guinea pig conjugated with Alexa 488, 568 and 647 (1:1000, Molecular Probes, Eugene, Oregon, USA).

## Data Availability

The datasets supporting the conclusions of this article are included within the article. Materials are available from the corresponding author on reasonable request.

## Abbreviations

DNA: Desoxyribonucleic acid
DSHB: Developmental Studies Hybridoma Bank
VDRC: Vienna Drosophila Resource Center

## References

[1] Goodman CS, Doe CQ, Bate M, Martinez-Arias A (1993). Embryonic development of the *Drosophila* central nervous system. The development of *Drosophila*.

[2] Younossi-Hartenstein A, Nassif C, Green P, Hartenstein V (1996). Early neurogenesis of the *Drosophila* brain. J Comp Neurol.

[3] Urbach R, Technau GM (2003). Molecular markers for identified neuroblasts in the developing brain of *Drosophila*. Development..

[4] Truman JW, Schuppe H, Shepherd D, Williams DW (2004). Developmental architecture of adult-specific lineages in the ventral CNS of *Drosophila*. Development..

[5] Doe CQ (2008). Neural stem cells: balancing self-renewal with differentiation. Development..

[6] Bello BC, Izergina N, Caussinus E, Reichert H (2008). Amplification of neural stem cell proliferation by intermediate progenitor cells in *Drosophila* brain development. Neural Dev.

[7] Boone JQ, Doe CQ (2008). Identification of *Drosophila* type II neuroblast lineage containing transit amplifying ganglion mother cells. Dev Neurobiol.

[8] Bowman SK, Rolland V, Betschinger J, Kinsey KA, Emery G, Knoblich JA (2008). The tumor suppressors brat and numb regulate transit-amplifying neuroblast lineages in *Drosophila*. Dev Cell.

[9] Walsh KT, Doe CQ (2017). *Drosophila* embryonic type II neuroblasts: origin, temporal patterning, and contribution to the adult central complex. Development..

[10] Álvarez J-A, Díaz-Benjumea FJ (2018). Origin and specification of type II neuroblasts in the *Drosophila* embryo. Development..

[11] Homem CCF, Knoblich JA (2012). *Drosophila* neuroblasts: a model for stem cell biology. Development..

[12] Sousa-Nunes R, Chen LY, Gould AP (2010). Regulating neural proliferation in the *Drosophila* CNS. Curr Opin Neurobiol.

[13] Green P, Hartenstein AY, Hartenstein V (1993). The embryonic development of the *Drosophila* visual system. Cell Tissue Res.

[14] Nériec N, Desplan C (2016). From the eye to the brain: development of the *Drosophila* visual system. Curr Top Dev Biol.

[15] Ngo KT, Andrade I, Hartenstein V (2017). Spatio-temporal pattern of neuronal differentiation in the *Drosophila* visual system: a user’s guide to the dynamic morphology of the developing optic lobe. Dev Biol.

[16] Pfeiffer K, Homberg U (2014). Organization and functional roles of the central complex in the insect brain. Annu Rev Entomol.

[17] Curt JR, Salmani BY, Thor S (2019). Anterior CNS expansion driven by brain transcription factors. eLife..

[18] Weng M, Golden KL, Lee C-Y (2010). dFezf/earmuff maintains the restricted developmental potential of intermediate neural progenitors in *Drosophila*. Dev Cell.

[19] Strecker TR, Kongsuwan K, Lengyel JA, Merriam JR (1986). The zygotic mutant *tailless* affects the anterior and posterior ectodermal regions of the *Drosophila* embryo. Dev Biol.

[20] Reim I, Lee H-H, Frasch M (2003). The T-box-encoding Dorsocross genes function in amnioserosa development and the patterning of the dorsolateral germ band downstream of Dpp. Development..

[21] Simeone A, D’Apice MR, Nigro V, Casanova J, Graziani F, Acampora D (1994). *Orthopedia*, a novel homeobox-containing gene expressed in the developing CNS of both mouse and *Drosophila*. Neuron..

[22] Hildebrandt K, Bach N, Kolb D, Walldorf U (2020). The homeodomain transcription factor Orthopedia is involved in development of the *Drosophila* hindgut. Hereditas..

[23] Eggert T, Hauck B, Hildebrandt N, Gehring WJ, Walldorf U (1998). Isolation of a *Drosophila* homolog of the vertebrate homeobox gene *Rx* and its possible role in brain and eye development. Proc Natl Acad Sci U S A.

[24] Davis RJ, Tavsanli BC, Dittrich C, Walldorf U, Mardon G (2003). Drosophila retinal homeobox (drx) is not required for establishment of the visual system, but is required for brain and clypeus development. Dev Biol.

[25] Walldorf U, Kiewe A, Wickert M, Ronshaugen M, McGinnis W (2000). *Homeobrain*, a novel paired-like homeobox gene is expressed in the *Drosophila* brain. Mech Dev.

[26] Kolb D, Kaspar P, Klöppel C, Walldorf U (2021). The *Drosophila* homeodomain transcription factor Homeobrain is involved in the formation of the embryonic protocerebrum and the supraesophageal brain commissure. Cells Dev.

[27] Guo X, Yin C, Yang F, Zhang Y, Huang H, Wang J (2019). The cellular diversity and transcription factor code of *Drosophila* enteroendocrine cells. Cell Rep.

[28] Graveley BR, Brooks AN, Carlson JW, Duff MO, Landolin JM, Yang L (2010). The developmental transcriptome of *Drosophila melanogaster*. Nature..

[29] Bayraktar OA, Doe CQ (2013). Combinatorial temporal patterning in progenitors expands neural diversity. Nature..

[30] Li X, Erclik T, Bertet C, Chen Z, Voutev R, Venkatesh S (2013). Temporal patterning of *Drosophila* medulla neuroblasts controls neural fates. Nature..

[31] Suzuki T, Kaido M, Takayama R, Sato M (2013). A temporal mechanism that produces neuronal diversity in the *Drosophila* visual center. Dev Biol.

[32] Grossniklaus U, Pearson RK, Gehring WJ (1992). The *Drosophila* sloppy paired locus encodes two proteins involved in segmentation that show homology to mammalian transcription factors. Genes Dev.

[33] Konstantinides N, Rossi A, Escobar A, Dudragne L, Chen Y-C, Tran T, et al. A comprehensive series of temporal transcriptions factors in the fly visual system. BioRxiv. 2021. doi: https://doi.org/10.1101/2021.06.13.448242.

[34] Kvon EZ (2015). Using transgenic reporter assays to functionally characterize enhancers in animals. Genomics..

[35] Pfeiffer BD, Jenett A, Hammonds AS, Ngo T-TB, Misra S, Murphy C (2008). Tools for neuroanatomy and neurogenetics in *Drosophila*. Proc Natl Acad Sci U S A.

[36] Jenett A, Rubin GM, Ngo T-TB, Shepherd D, Murphy C, Dionne H (2012). A GAL4-driver line resource for *Drosophila* neurobiology. Cell Rep.

[37] Jory A, Estella C, Giorgianni MW, Slattery M, Laverty TR, Rubin GM (2012). A survey of 6,300 genomic fragments for cis-regulatory activity in the imaginal discs of *Drosophila melanogaster*. Cell Rep.

[38] Manning L, Heckscher ES, Purice MD, Roberts J, Bennett AL, Kroll JR (2012). A resource for manipulating gene expression and analyzing cis-regulatory modules in the *Drosophila* CNS. Cell Rep.

[39] Brand AH, Perrimon N (1993). Targeted gene expression as a means of altering cell fates and generating dominant phenotypes. Development..

[40] Rong YS, Golic KG (2000). Gene targeting by homologous recombination in *Drosophila*. Science..

[41] Rong YS, Golic KG (2001). A targeted gene knockout in *Drosophila*. Genetics..

[42] Gong WJ, Golic KG (2003). Ends-out, or replacement, gene targeting in *Drosophila*. Proc Natl Acad Sci U S A.

[43] Gratz SJ, Cummings AM, Nguyen JN, Hamm DC, Donohue LK, Harrison MM (2013). Genome engineering of *Drosophila* with the CRISPR RNA-guided Cas9 nuclease. Genetics..

[44] Sternberg SH, Redding S, Jinek M, Greene EC, Doudna JA (2014). DNA interrogation by the CRISPR RNA-guided endonuclease Cas9. Nature..

[45] Bassett AR, Tibbit C, Ponting CP, Liu J-L (2013). Highly efficient targeted mutagenesis of *Drosophila* with the CRISPR/Cas9 system. Cell Rep.

[46] Kondo S, Ueda R (2013). Highly improved gene targeting by germline-specific Cas9 expression in *Drosophila*. Genetics..

[47] Yu Z, Ren M, Wang Z, Zhang B, Rong YS, Jiao R (2013). Highly efficient genome modifications mediated by CRISPR/Cas9 in *Drosophila*. Genetics..

[48] Jan LY, Jan YN (1992). Antibodies to horseradish peroxidase as specific neuronal markers in *Drosophila* and in grasshopper embryos. Proc Natl Acad Sci U S A.

[49] Barthalay Y, Hipeau-Jacquotte R, de la Escalera S, Jiménez F, Piovant M (1990). *Drosophila* neurotactin mediates heterophilic cell adhesion. EMBO J.

[50] Egger B, Boone JQ, Stevens NR, Brand AH, Doe CQ (2007). Regulation of spindle orientation and neural stem cell fate in the *Drosophila* optic lobe. Neural Dev.

[51] Meinertzhagen IA, Hanson TE. The development of the optic lobe: Bate, Martinez Arias; 1993. p. 1363–491.

[52] Wagh DA, Rasse TM, Asan E, Hofbauer A, Schwenkert I, Dürrbeck H (2006). Bruchpilot, a protein with homology to ELKS/CAST, is required for structural integrity and function of synaptic active zones in *Drosophila*. Neuron..

[53] Bayraktar OA, Boone JQ, Drummond ML, Doe CQ (2010). *Drosophila* type II neuroblast lineages keep Prospero levels low to generate large clones that contribute to the adult brain central complex. Neural Dev.

[54] Xiao Q, Komori H, Lee CY (2012). Klumpfuss distinguishes stem cells from progenitor cells during asymmetric neuroblast division. Development..

[55] Klöppel C, Hildebrandt K, Kolb D, Fürst N, Bley I, Karlowatz RJ (2021). Functional analysis of enhancer elements regulating the expression of the *Drosophila* homeodomain transcription factor DRx by gene targeting. Hereditas..

[56] Bier E, Vaessin H, Younger-Shepherd S, Jan LY, Jan YN (1992). *Deadpan*, an essential pan-neural gene in *Drosophila*, encodes a helix-loop-helix protein similar to the hairy gene product. Genes Dev.

[57] Ikeshima-Kataoka H, Skeath JB, Nabeshima Y, Doe CQ (1997). Miranda directs Prospero to a daughter cell during *Drosophila* asymmetric divisions. Nature..

[58] Berger C, Renner S, Lüer K, Technau GM (2007). The commonly used marker ELAV is transiently expressed in neuroblasts and glial cells in the *Drosophila* embryonic CNS. Dev Dyn.

[59] Campos AR, Rosen DR, Robinow SN, White K (1987). Molecular analysis of the locus *elav* in *Drosophila melanogaster*: a gene whose embryonic expression is neural specific. EMBO J.

[60] Robinow S, White K (1991). Characterization and spatial distribution of the ELAV protein during *Drosophila melanogaster* development. J Neurobiol.

[61] Campbell G, Göring H, Lin T, Spana E, Andersson S, Doe CQ (1994). RK2, a glial-specific homeodomain protein required for embryonic nerve cord condensation and viability in *Drosophila*. Development..

[62] Halter DA, Urban J, Rickert C, Ner SS, Ito K, Travers AA (1995). The homeobox gene *repo* is required for the differentiation and maintenance of glia function in the embryonic nervous system of *Drosophila melanogaster*. Development..

[63] Bertet C, Li X, Erclik T, Cavey M, Wells B, Desplan C (2014). Temporal patterning of neuroblasts controls notch-mediated cell survival through regulation of hid or reaper. Cell..

[64] Plavicki JS, Squirrell JM, Eliceiri KW, Boekhoff-Falk G (2016). Expression of the *Drosophila* homeobox gene, distal-less, supports an ancestral role in neural development. Dev Dyn.

[65] Baena-Lopez LA, Alexandre C, Mitchell A, Pasakarnis L, Vincent J-P (2013). Accelerated homologous recombination and subsequent genome modification in *Drosophila*. Development..

[66] Egger B, Gold KS, Brand AH (2010). Notch regulates the switch from symmetric to asymmetric neural stem cell division in the *Drosophila* optic lobe. Development..

[67] Younossi-Hartenstein A, Nguyen B, Shy D, Hartenstein V (2006). Embryonic origin of the *Drosophila* brain neuropile. J Comp Neurol.

[68] Sprecher S, Reichert H, Hartenstein V (2007). Gene expression patterns in primary neuronal clusters of the *Drosophila* embryonic brain. Gene Expr Patterns.

[69] Larsen C, Shy D, Spindler SR, Fung S, Pereanu W, Younossi-Hartenstein A (2009). Patterns of growth, axonal extension and axonal arborization of neuronal lineages in the developing *Drosophila* brain. Dev Biol.

[70] Nassif C, Noveen A, Hartenstein V (1998). Embryonic development of the *Drosophila* brain. I. Pattern of pioneer tracts. J Comp Neurol.

[71] Tang JLY, Hakes AE, Krautz R, Suzuki T, Contreras EG, Fox PM, et al. NanoDam identifies novel temporal transcription factors conserved between the *Drosophila* central brain and visual system. BioRxiv. 2021. 10.1101/2021.06.07.447332.

[72] Li MA, Alls JD, Avancini RM, Koo K, Godt D (2003). The large Maf factor traffic jam controls gonad morphogenesis in *Drosophila*. Nat Cell Biol.

[73] Finkelstein R, Smouse D, Capaci TM, Spradling AC, Perrimon N (1990). The *orthodenticle* gene encodes a novel homeo domain protein involved in the development of the *Drosophila* nervous system and ocellar visual structures. Genes Dev.

[74] Apitz H, Salecker I (2015). A region-specific neurogenesis mode requires migratory progenitors in the *Drosophila* visual system. Nat Neurosci.

[75] Kvon EZ, Kazmar T, Stampfel G, Yánez-Cuna JO, Pagani M, Schernhuber (2014). Genome-scale functional characterization of *Drosophila* developmental enhancers in vivo. Nature..

[76] Perry MW, Boettiger AN, Bothma JP, Levine M (2010). Shadow enhancers foster robustness of *Drosophila* gastrulation. Curr Biol.

[77] Cannavò E, Khoueiry P, Garfield DA, Geeleher P, Zichner T, Gustafson EH (2016). Shadow enhancers are pervasive features of developmental regulatory networks. Curr Biol.

[78] El-Sherif E, Levine M (2016). Shadow enhancers mediate dynamic shifts of gap gene expression in the *Drosophila* embryo. Curr Biol.

[79] Therianos S, Leuzinger S, Hirth F, Goodman CS, Reichert H (1995). Embryonic development of the *Drosophila* brain: formation of commissural and descending pathways. Development..

[80] Capecchi MR (1989). Altering the genome by homologous recombination. Science..

[81] Zákány J, Gérard M, Favier B, Duboule D (1997). Deletion of a HoxD enhancer induces transcriptional heterochrony leading to transposition of the sacrum. EMBO J.

[82] Leighton PA, Saam JR, Ingram RS, Stewart CL, Tilghman SM (1995). An enhancer deletion affects both H19 and Igf2 expression. Genes Dev.

[83] Rogers WA, Goyal Y, Yamaya K, Shvartsman SY, Levine MS (2017). Uncoupling neurogenic gene networks in the *Drosophila* embryo. Genes Dev.

[84] Dunipace L, Ákos Z, Stathopoulos A (2019). Coacting enhancers can have complementary functions within gene regulatory networks and promote canalization. PLoS Genet.

[85] Hildebrandt K, Kübel S, Minet M, Fürst N, Klöppel C, Steinmetz E (2021). Enhancer analysis of the *Drosophila* zinc finger transcription factor earmuff by gene targeting. Hereditas..

[86] Osterwalder M, Barozzi I, Tissières V, Fukuda-Yuzawa Y, Mannion BJ, Afzal SY (2018). Enhancer redundancy provides phenotypic robustness in mammalian development. Nature..

[87] Hoskins RA, Nelson CR, Berman BP, Laverty TR, George RA, Ciesiolka L (2000). A BAC-based physical map of the major autosomes of *Drosophila melanogaster*. Science..

